# Mucosal immune alterations at the early onset of tissue destruction in chronic obstructive pulmonary disease

**DOI:** 10.3389/fimmu.2023.1275845

**Published:** 2023-10-17

**Authors:** Charlotte de Fays, Vincent Geudens, Iwein Gyselinck, Pieterjan Kerckhof, Astrid Vermaut, Tinne Goos, Marie Vermant, Hanne Beeckmans, Janne Kaes, Jan Van Slambrouck, Yousry Mohamady, Lynn Willems, Lucia Aversa, Emanuela E. Cortesi, Charlotte Hooft, Gitte Aerts, Celine Aelbrecht, Stephanie Everaerts, John E. McDonough, Laurens J. De Sadeleer, Sophie Gohy, Jerome Ambroise, Wim Janssens, Laurens J. Ceulemans, Dirk Van Raemdonck, Robin Vos, Tillie L. Hackett, James C. Hogg, Naftali Kaminski, Ghislaine Gayan-Ramirez, Charles Pilette, Bart M. Vanaudenaerde

**Affiliations:** ^1^ Pole of Pneumology, ENT, and Dermatology, Institute of Experimental and Clinical Research, Université Catholique de Louvain, Brussels, Belgium; ^2^ Laboratory of Respiratory Diseases and Thoracic Surgery, BREATHE, Department of CHROMETA, KULeuven, Leuven, Belgium; ^3^ Section of Pulmonary, Critical Care, and Sleep Medicine, Yale University School of Medicine, New Haven, CT, United States; ^4^ Department of Pneumology, Cliniques Universitaires Saint-Luc, Brussels, Belgium; ^5^ Centre de Technologies Moléculaires Appliquées, Institute of Experimental and Clinical Research, Université Catholique de Louvain, Brussels, Belgium; ^6^ Centre for Heart Lung Innovation, St Paul’s Hospital, Vancouver, BC, Canada

**Keywords:** lung mucosal immunity, chronic obstructive pulmonary disease, airway inflammation, lung tissue destruction, emphysema severity

## Abstract

**Rationale:**

COPD is characterized by chronic airway inflammation, small airways changes, with disappearance and obstruction, and also distal/alveolar destruction (emphysema). The chronology by which these three features evolve with altered mucosal immunity remains elusive. This study assessed the mucosal immune defense in human control and end-stage COPD lungs, by detailed microCT and RNA transcriptomic analysis of diversely affected zones.

**Methods:**

In 11 control (non-used donors) and 11 COPD (end-stage) explant frozen lungs, 4 cylinders/cores were processed per lung for microCT and tissue transcriptomics. MicroCT was used to quantify tissue percentage and alveolar surface density to classify the COPD cores in mild, moderate and severe alveolar destruction groups, as well as to quantify terminal bronchioles in each group. Transcriptomics of each core assessed fold changes in innate and adaptive cells and pathway enrichment score between control and COPD cores. Immunostainings of immune cells were performed for validation.

**Results:**

In mildly affected zones, decreased defensins and increased mucus production were observed, along CD8+ T cell accumulation and activation of the IgA pathway. In more severely affected zones, CD68+ myeloid antigen-presenting cells, CD4+ T cells and B cells, as well as MHCII and IgA pathway genes were upregulated. In contrast, terminal bronchioles were decreased in all COPD cores.

**Conclusion:**

Spatial investigation of end-stage COPD lungs show that mucosal defense dysregulation with decreased defensins and increased mucus and IgA responses, start concomitantly with CD8+ T-cell accumulation in mild emphysema zones, where terminal bronchioles are already decreased. In contrast, adaptive Th and B cell activation is observed in areas with more advanced tissue destruction. This study suggests that in COPD innate immune alterations occur early in the tissue destruction process, which affects both the alveoli and the terminal bronchioles, before the onset of an adaptive immune response.

## 1 Introduction

Chronic Obstructive Pulmonary Disease (COPD) is a highly prevalent lung disease (1) and the third leading cause of death worldwide (2), which is caused by longstanding exposure to inhaled toxics (mainly cigarette smoke as well as biomass, occupational, air pollution) and genetic predisposition (3). COPD pathology includes destruction of the alveolar walls referred to as emphysema, large airway inflammation, and small airway (< 2mm in diameter) obstruction and destruction (4), with all these features heterogeneously distributed in the different zones of the lung parenchyma. The course of the disease is marked by the occurrence of acute worsening of symptoms called exacerbations, most of which are caused by viral or bacterial infections (5), which are likely promoted by impaired frontline defense mechanisms such as mucociliary clearance.

The mucosal barrier in the lung is barely one cell-layer thick epithelial surface and is one of the largest in the body (6, 7). Mucosal defense of the lung includes the physical barrier of the epithelium and its intercellular junctions, mucociliary clearance, epithelial production of antimicrobial peptides (AMP) and oxidative agents as well as transport of IgA antibodies, as well as professional innate and adaptive immune cells, most of which are altered in COPD (8). For instance some COPD airways display a deficiency in IgA at the epithelial-lining surface due to downregulation of its transepithelial transporter, the polymeric immunoglobulin receptor (pIgR) (9, 10), or in the production of AMP following cigarette smoke exposure (11). It is thought that this loss of mucosal homeostasis in COPD leads to immune activation reflected by the recruitment and accumulation of innate and adaptive immune cells, as well as the formation of tertiary lymphoid follicles around small airways (12). Interestingly, IgA-producing B cells are selectively increased within airway associated lymphoid follicles in COPD (13). It remains however unclear whether, in COPD, immune activation and mucosal immunity alterations take place before or after alveolar and terminal bronchioles destruction. This has been addressed by combining microCT imaging and tissue RNA profiling in order to assess mucosal immune features and terminal bronchioles count in zones with diverse levels of alveolar tissue destruction in end-stage COPD lungs, as compared to control lungs.

## 2 Materials and methods

### 2.1 Study material

From our UZ/KULeuven biobank (S51577), 11 COPD explant lungs (collected at the time of lung transplantation) and 11 discarded donor lungs that were declined by the transplant surgeon due to a non-pulmonary reason were used for this study. Lungs were rejected for transplantation due to kidney tumor (n=1), emboli (n=4), contusion (n=2), recipient dying (n=2), beginning fibrosis (n=1), rupture of an artery (n=1). Written informed consent was provided by all patients awaiting for lung transplantation for end-stage COPD. The collection of unused donor lungs for research purposes was made in accordance with the Belgian law. The study was approved by the Medical Ethics Board of University Hospitals Leuven, Belgium (S52174).

### 2.2 Lung tissue processing and sampling

**Figure 1:**
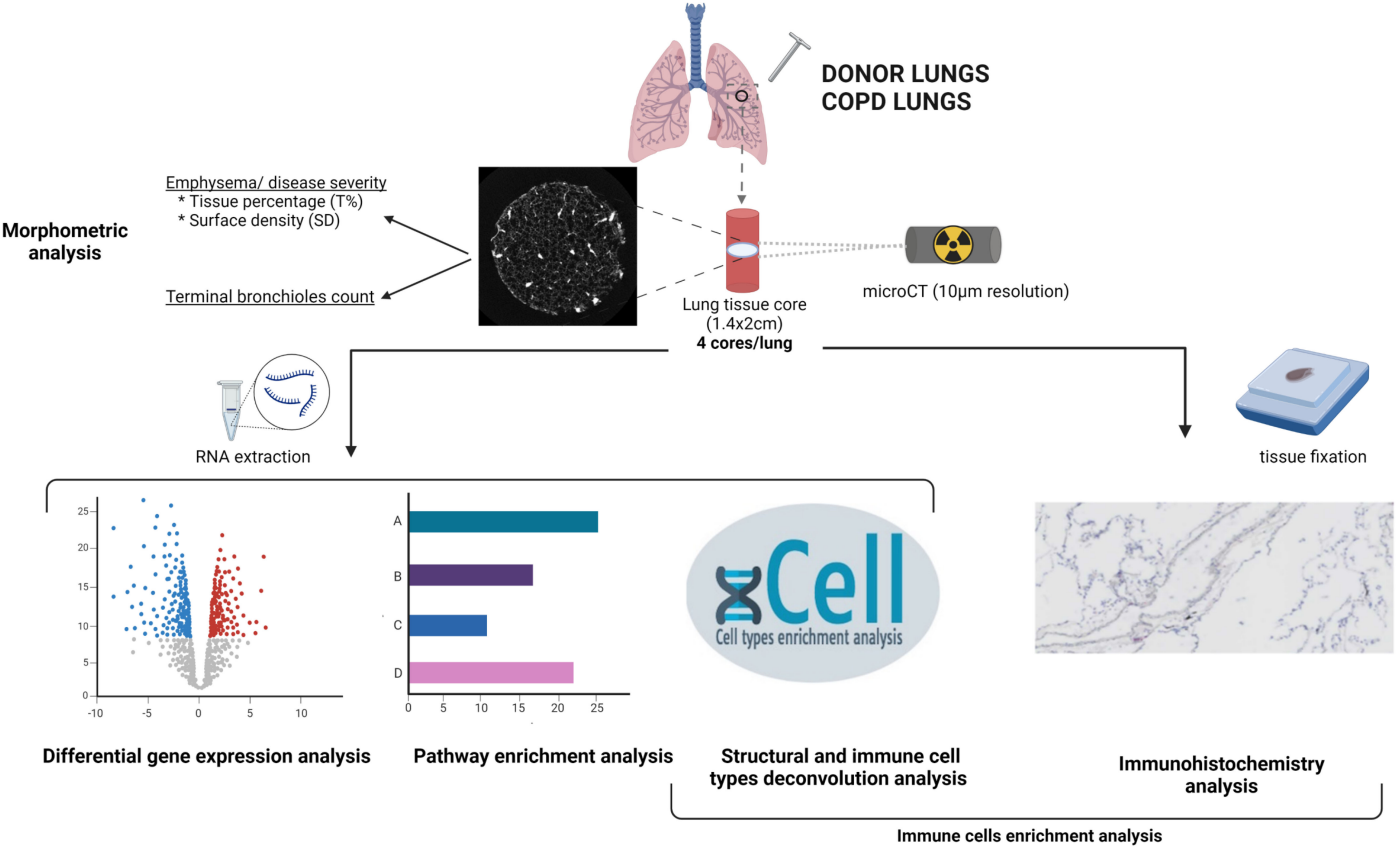
Schematic overview of the methodology. Unused donor and COPD explant lungs were inflated at total lung capacity and frozen. Four cores were sampled from each lung, and a high resolution microCT of each core was performed, for morphometric analysis. One piece of each core was used for RNA extraction. RNAseq data were used for differential gene expression analysis, pathway enrichment analysis and cellular deconvolution enrichment scores analysis. Another piece of each core was used for cell type immunostainings. Created with *BioRender.com* .

Study design is summarized in **Figure 1**. Lungs specimens were processed in a standard way as described before (4). In brief, the main stem bronchus of each lung was cannulated and lungs were inflated to a transpulmonary pressure of 30 cm H_2_O at total lung capacity, then deflated to 10 cm H_2_O and held at that pressure to be subsequently frozen in liquid nitrogen vapors and stored at -80°C. High resolution computed tomography (HRCT) scan was obtained of all frozen lungs that were then cut into contiguous 2-cm-thick slices from lung apex to base. About 10 cores (diameter 1.4cm; height 2 cm) of frozen tissue were systematically obtained from each slices using a sharpened steel cylinder. Four cores per lung were randomly selected for further analysis, including two cores from the apical slices and two cores from the basal slices in order to reflect the spatial heterogeneity in the lung. Unused donor lung tissue cores were sampled in the same way, with strict avoidance of areas with suspicion of any abnormality.

### 2.3 MicroCT and morphological analysis

**Figure 2:**
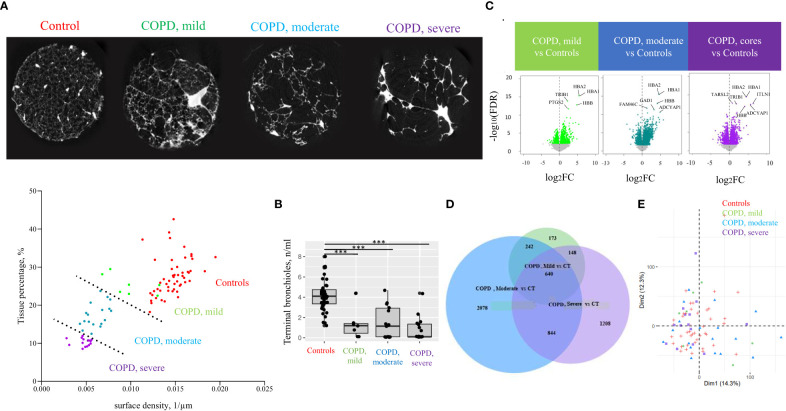
Classification of lung tissue cores according to emphysema severity. **(A)** Cores tissue percentage and surface density were determined based on microCT and used to define emphysema severity groups among COPD cores, separated by a dashed line (lower panel): mild COPD cores, moderate COPD cores and severe COPD cores, in addition to control cores (CT). A transversal section microCT of one representative core per group is presented (upper panel). Each point corresponds to individual core data. **(B)** Terminal bronchioles (TB) count in controls and COPD cores. Each point corresponds to individual core data, ***p<0.0005 *vs* controls (CT) by a linear mixed effects regression models including cores disease subgroups, age, and gender as fixed effect and patient as random effect **(C)** Volcano plots of differential gene expression data in mild COPD cores, moderate COPD cores and severe COPD cores as compared with controls. Top significant genes are labeled. Each point corresponds to individual core data. **(D)** PCA analysis with component 1 and 2 plotted. Each point corresponds to individual core data. **(E)** Venn diagram showing numbers of differentially expressed genes in each cores group comparison with controls.

MicroCT was used to scan the frozen cores at a resolution of 10 µm (Bruker Skyscan 1172 microCT device, Bruker, Kontich, Belgium) on a cooling stage that preserves the cores at -30°C. MicroCT images were reconstructed with NRecon (Bruker, Belgium) and analyzed using CTan to measure surface area, tissue volume and core volume, with a manual thresholding for differentiating tissue from air (**Figure 2A**). The ratio of surface area by core volume was defined as alveolar surface density (ASD) and the ratio of tissue volume by core volume as tissue percentage (T%).

Clustering of the COPD cores according to the extent of emphysema, based on tissue percentage and surface density, resulted in three emphysema severity cores groups, namely COPD, mild (n=7), COPD, moderate (n=18) and COPD, severe cores (n=14), as well as controls (n= 43) (**Figure 2A**). We excluded three cores from one COPD lung and one core from another COPD lung due to structural abnormalities (hyperdensities representing >50% of total core), and one sample from one control lung and another from one COPD lung for invalid RNA transcriptomic data.

In order to count terminal bronchioles in each emphysema severity cores groups, terminal bronchioles were identified on microCT scans as the last segment of airways before the transition to respiratory bronchioles, and the number of terminal bronchioles in each core was manually counted. Terminal bronchioles counts were compared between COPD and the control cores groups using linear mixed effects regression models including cores disease subgroups, age, and gender as fixed effect and patient as random effect. A p-value <0.05 was considered significant.

After microCT, all cores were divided in multiple pieces, with one used for RNA extraction and transcriptome analysis, and another one embedded for immunohistochemistry.

### 2.4 RNA processing and transcriptomics data analysis

RNA extraction from each core was performed using miRNeasy micro kit (Quiagen), as previously described (14). Samples with RIN>5 were included in the study. Twenty nanograms of RNA per core was used to prepare cDNA libraries using Ion AmpliSeq Transcriptome Human Gene Expression kit according to the manufacturer’s protocol. The fastq files data were then mapped on the human genome (UCSC hg38) using a two stages strategy using first splicing-aware mapping tool STAR and then Bowtie2 for the remaining unmapped reads.

The read count expression matrix was analyzed using the edger v3.40.2 Bioconductor package (15) in order to detect differentially expressed genes (DEGs). Briefly, a filtering strategy was first applied in order to keep genes having sufficiently large counts to be retained in statistical analyses. Scaling factors were then computed with the trimmed mean of M-values (TMM) method. A quasi-likelihood negative binomial generalized log-linear model was built on the resulting data. In addition to the variable of interest (e.g. COPD cores subgroups and control), the age and gender of the patients were explicitly incorporated in the models in order to correct for the potential confounding effects of these factors. Based on the resulting gene expression modulations, Gene Set Enrichment Analysis (GSEA) was performed using the WebGestaltR v0.4.4 (16–18) Bioconductor packages on the KEGG database (19–21).

In parallel, immune and structural cell subtype enrichment scores were computed from FKPM normalized gene expression data using xCell R package. This package performs gene-signature based cell type deconvolution analysis of 64 immune and stromal cell types (22). Signature genes for each cell types were validated using in-silico stimulations and cytometry immunophenotyping during package development. Fold changes of immune and structural cell subtypes xCell enrichment scores between controls cores group and each COPD severity cores groups was then calculated using linear mixed effects regression models including cores disease subgroups, age, and gender as fixed effect and patient as random effect.

P-values obtained during transcriptomic analysis were adjusted for multiple testing using the Benjamini-Hochberg correction with a false discovery rate (FDR) of 0.1 considered significant.

### 2.5 Immunohistochemistry

One piece of each core was vacuum embedded in OCT (Sakura), and frozen sections (8 µm) were obtained. Stainings for CD8 (M7103, Dako Hervelee, Belgium), CD4 (M7310, Dako Heverlee, Dako Heverlee, Belgium), CD68 (M0876, Dako Heverlee, Belgium), CD20 (M0755, Dako Heverlee, Belgium), and collagen 1 (ab34710, Abcam, USA) were performed, using an AEC chromogen (K3464, Dako Heverlee, Belgium). Slides were scanned (Aperio Digital pathology slide scanner, Leica Biosystem Inc., Canada), and images were generated using QuPath. Tresholding of stained positive area and tissue area was performed using the ImageJ software. Results were expressed as the percentage of stained positive area on total tissue area, as referred to as staining index. Staining indices were compared between COPD emphysema severity and control cores group using mixed effects regression model including age and gender as fixed effects and patient as random effect. A p-value <0.05 was considered significant.

## 3 Results

### 3.1 Patients characteristics and terminal bronchioles count in COPD lung tissue

Patient demographics of the 11 COPD and 11 control lungs is shown in **Table 1**. Terminal bronchioles were decreased in mild (estimate -2.5, p<0.001), moderate (estimate -2.8, p<0.001), and severe (estimate -3.4 p<0.001) COPD cores, as compared with controls (**Figure 2B**).

**Table 1:** Patients characteristics.

	Controls	COPD	p-values
**Subjects, n**	11	11	
**Age, years**	48 (23)	60 (3)	ns
**Age, range**	16-72	48-61	
**Male, n (%)**	9 (81)	5 (45)	
**Height, cm**	175 (15)	163 (22)	ns
**Weight, kg**	80 (25)	60 (17)	ns
**BMI, kg/m²**	25 (4)	21 (8)	ns
**FEV1, L**	NA	0.8 (0.5)	
**FEV1, % predicted**	NA	31 (11)	
**FVC, L**	NA	2.0 (0.4)	
**FVC, % predicted**	NA	66 (33)	
**FEV1/FVC**	NA	0.4 (0.1)	
**DLCO, % predicted**	NA	33 (14)	
**Smoking, pack-year**	NA	35 (50)	
**Inhalated corticosteroids, n (%)** **medium dose** **high dose**	NA NA	3 (27) 8 (72)	

Results are presented as n (%) or median (IQR). % predicted of FEV1 and FVC was calculated on ECSC equations before 2012 and on GLI equations from 2012. NA, not applicable; BMI, body mass index; FEV1, forced expiratory volume in 1 second; FVC, forced vital capacity; FEV1/FVC, Tiffeneau index; DLCO, diffusion capacity of the lung for carbon monoxide. P-value >0.05 was considered non-significant (ns) (Wilcoxon-Mann-Withney test).

### 3.2 Transcriptomic analysis

**Figure 3:**
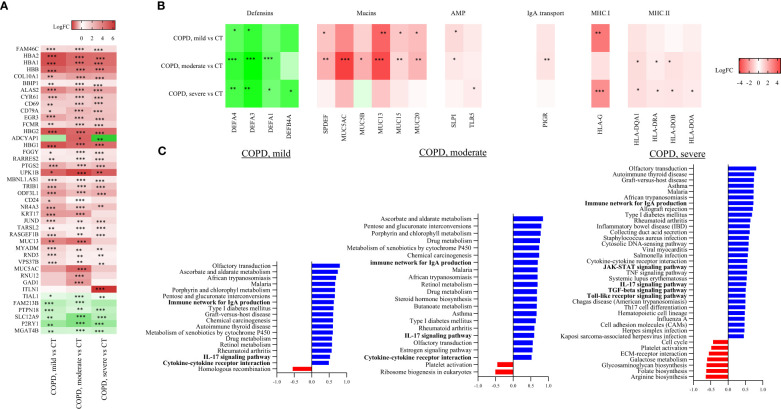
Cores RNA-seq based transcriptome analysis. **(A)** Top differentially expressed genes in mild COPD cores, moderate COPD cores and severe COPD cores as compared to control cores (CT). *FDR<0.1, **FDR<0.01, ***FDR<0.001 *vs* controls by a multiple linear regression test including age and gender as variables. **(B)** Differential expression data of genes involved in antigen presentation and mucosal immunity. * FDR<0.1, **FDR<0.01, ***FDR<0.001 *vs* controls using a multiple linear regression test including age and gender as variables. **(C)** Significantly up- and downregulated pathways in mild COPD cores (left panel), moderate COPD cores (middle panel) and severe COPD cores (right panel) as compared with controls. * FDR<0.1, **FDR<0.01, ***FDR<0.001 versus controls by a multiple linear regression test including age and gender as variables. AMP, antimicrobial peptides; MHC, Major histocompatibility complex.

Among the 17 203 genes analyzed, respectively 1203, 3804 and 2840 genes were differentially expressed in the mild, moderate and severe COPD cores, respectively, as compared with controls (**Figure 2C**). 882 genes were upregulated in both mild and moderate COPD cores, 1484 genes in both moderate and severe COPD cores, 788 in both mild and severe COPD cores, and 640 genes were upregulated in mild, moderate and severe COPD cores (**Figure 2D**). Principal component analysis of all gene expression data has been performed, showing a dispersion of the cores of our four sample groups (**Figure 2E**). Genes of hemoglobins as well as FAM46C have been identified as the top differentially expressed genes (**Figure 3A**).

#### 3.2.1 Mucosal defense alterations and antigen presentation in COPD areas

Differential expression analysis of genes (**Table 2**, **Figure 3B**) involved in mucosal innate defense in COPD showed a progressively decreasing expression of several defensins, respectively DEFA4 and DEFA3, when comparing respectively mild, moderate and severe COPD cores with control cores. Other defensins, such as DEFA1 and DEFB4A also decreased in moderate and severe COPD cores, without reaching statistical significance in mild COPD cores as compared with controls. In contrast, MUC13 was increased in mild COPD cores as compared with controls along other mucins as well as the transcription factor SPDEF. All those genes were further increased in moderate COPD cores, as compared with controls. Expression of cathelicidin (CAMP) and TLRs (TLR5, 7, 8 and 9) was not significantly altered in COPD cores, while secretory leucocyte protease inhibitor (SLPI) expression was increased in mild COPD cores, as well as in moderate COPD cores as compared with controls. A trend to increase of PIGR was also observed in mild COPD cores as compared with controls, with an increase in moderate COPD cores as compared with controls. Finally, several collagen genes were increased, with COL10A1 increased in all COPD groups compared to controls, while COL7A1 and COL17A1 were increased in moderate and severe COPD cores as compared with controls (**Table 2**, **Figure 3B**).

**Table 2:** Comparison of genes expression and pathways enrichment scores involved in innate mucosal defenses and (IgA-mediated) adaptive immunity.

	COPD, mild cores vs CT		COPD, moderate cores vs CT		COPD, severe cores vs CT	
Genes	LogFC	adj p-value	LogFC	adj p-value	LogFC	adj p-value
Defensins						
**DEFA4**	-2.66	**0.06**	-3.34	**<0.001**	-3.02	**0.002**
**DEFA3**	-3.34	**0.03**	-4.18	**<0.001**	-3.77	**0.001**
**DEFA1**	-2.28	0.16	-2.53	**0.003**	-2.3	**0.02**
**DEFB4A**	-2.97	0.11	-1.09	0.315	-3.04	**0.01**
Mucins						
**MUC5AC**	2.9	0.12	4.72	**<0.001**	1.71	0.29
**MUC5B**	1.14	0.49	2.02	**0.018**	-0.52	0.72
**SPDEF**	1.45	**0.09**	1.95	**0.001**	0.34	0.67
**MUC13**	3.82	**0.008**	4.44	**<0.001**	1.43	0.27
**MUC15**	1.4	**0.01**	1.02	**0.003**	0.525	0.25
**MUC20**	1.64	**0.06**	1.81	**0.001**	0.157	0.87
Antimicrobial peptides						
**SLPI**	0.91	**0.06**	0.58	**0.06**	0.27	0.52
**TLR5**	0.54	0.17	0.4	0.12	0.51	**0.07**
Genes involved in IgA-mediated adaptive immune response						
**PIGR**	0.64	0.16	0.68	**0.008**	0.13	0.76
MHC Class I genes						
**HLA-G**	3.33	**0.002**	0.756	0.345	2.76	**<0.001**
MHC Class II genes						
**HLA-DQA1**	0.69	0.26	0.86	**0.02**	0.84	**0.04**
**HLA-DRA**	0.39	0.30	0.47	**0.03**	0.60	**0.01**
**HLA-DOB**	0.15	0.82	0.70	**0.02**	0.65	**0.06**
**HLA-DOA**	0.39	0.38	0.56	0.03	0.50	**0.09**
Pathways	Enrichment score	adj p-value	Enrichment score	adj p-value	Enrichment score	adj p-value
**Immune network for IgA production**	0.63	**0.02**	0.70	**0.001**	0.73	**<0.001**
**IL-17 signaling pathway**	0.52	**0.08**	0.59	**0.02**	0.53	**0.02**
**Cytokine-Cytokine receptor interaction**	0.49	**0.06**	0.52	**0.09**	0.55	**<0.001**
**TGF-beta signaling pathway**	0.51	0.31	0.50	0.31	0.50	**0.04**
**Toll-like receptor signaling pathway**	0.44	0.39	0.20	0.99	0.50	**0.04**
**JAK-STAT signaling pathway**	0.48	0.13	0.41	0.87	0.55	**0.002**
**Th17 cell differentiation**	0.3	0.7	0.44	0.69	0.49	**0.04**

Results are presented as log2-transformed fold change (log2FC) of gene expression data and pathway enrichment scores between each COPD cores groups as compared with controls cores group and FDR adjusted p-value, calculated using a linear multiple regression analysis including gender and age as covariates. FDR<0.1 was considered significant. Statistically significant results are in bold.

Antigen presentation by major histocompatibility complex class 1 (MHCI), essential for CD8+ T-cell activation, and MHCII, needed for CD4+ T cells activation, were assessed through differential expression analysis of class I and class II MHC genes between each COPD and control groups. Among MHCI genes, HLA-G gene was increased in mild COPD cores and in severe COPD cores, while there was no difference in expression of other MHCI genes as compared with controls (**Table 2**, **Figure 3B**). Differential expression of MHCII genes showed an increase in several MHCII genes, namely HLA-DQA1, HLA-DRA, HLA-DOB and HLA-DOA, in either moderate or severe COPD cores or both compared with control cores, with no differences in mild COPD cores (**Table 2**, **Figure 3B**).

#### 3.2.2 Pathways involved in IgA production and adaptive immune response activation in COPD areas

GSEA analysis showed a significant increase of pathways involved in IgA production in mild, moderate and severe COPD cores compared with controls cores (**Table 2**, **Figure 3C**) (GSEA data analysis, **Table E3**). IL-17 signaling pathway was also significantly increased in mild, moderate and severe COPD cores, as compared with control cores.

In addition, an increase of signature genes involved in TGF-ß and Toll-like receptor signaling, as well as Th17 differentiation was observed in severe COPD *vs* control cores (**Tables 2**, **E3**, **Figure 3C**).

### 3.3 Immune cell types analysis

#### 3.3.1 Cellular RNA deconvolution analysis

**Figure 4:**
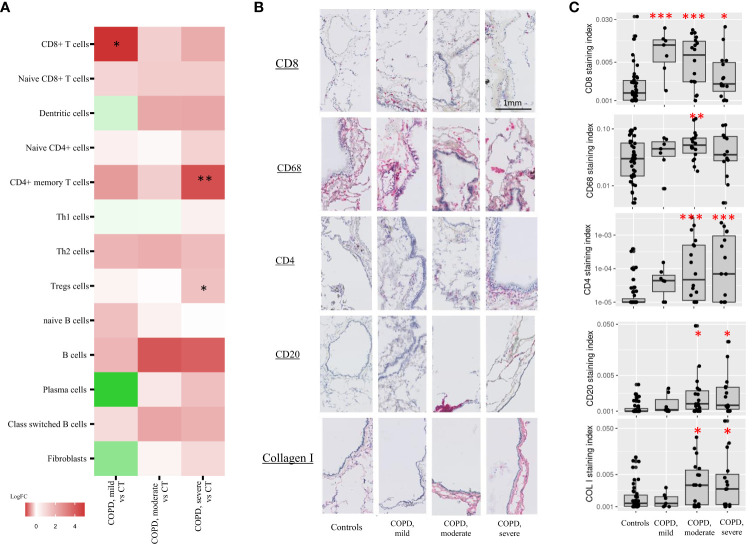
Immune cell types cores composition. **(A)** xCell enrichment scores of cell types involved in the adaptive immune response and fibroblasts in COPD, moderate and –severe cores as compared with controls cores (CT). Each point corresponds to individual core data, * FDR<0.1, **FDR<0.01, ***FDR<0.001 versus controls by a linear mixed effects regression models including cores disease subgroups, age, and gender as fixed effect and patient as random effect. **(B, C)** Adaptive immune cell types and fibroblasts immunostainings (log2-transformed staining index). Each point corresponds to individual core data, * p<0.05, **p<0.01, ***p<0.001 *vs* controls by a linear mixed effects regression models including cores disease subgroups, age, and gender as fixed effect and patient as random effect.

Cellular deconvolution enrichment scores in mild COPD cores compared with controls showed increased CD8+ T cells. CD8+ T cells deconvolution enrichment score was, in contrast, not increased in moderate and severe COPD cores as compared with controls (**Table 3**, **Figure 4A**).

**Table 3:** Enrichment scores in immune cell types comparison.

Cells	COPD, mild cores vs CT		COPD, moderate cores vs CT		COPD, severe cores vs CT	
	LogFC	FDR	LogFC	FDR	LogFC	FDR
**CD8+ T cells**	5.06	**0.031**	1.19	0.731	2.06	0.571
**Dentritic cells**	-0.27	0.939	2.13	0.561	2.19	0.517
**B cells**	1.82	0.858	4.17	0.113	3.91	0.156
**Class switched B cells**	0.87	0.897	2.21	0.202	1.93	0.343
**CD4+ memory T cells**	2.45	0.361	1.23	0.660	4.30	**0.004**
**Tregs cells**	0.280	0.908	0.06	0.978	1.53	**0.095**

Results are presented as log2-transformed fold change (log2FC) of xCell cell type enrichment scores between each COPD cores groups as compared with controls cores group and FDR adjusted p-value, calculated using a linear mixed model, including gender and age as fixed effect and patient as random effect. FDR<0.1 was considered significant. Statistically significant results are in bold.

In addition, xCell analysis revealed increased CD4+ memory T cells and regulatory T cells (Tregs) were observed in severe COPD cores *vs* control cores. Although not reaching statistical significance, increased B cells and class switch B-cells signature genes were also observed in moderate and severe COPD cores as compared with controls. No difference was observed in neutrophils in either COPD as compared with controls cores (**Figure E1**, **Table E2**). Finally, no difference in fibroblast signature genes was also observed in COPD cores compared to controls cores (**Table 3**, **Figure 4A**).

#### 3.3.2 Immunohistochemistry

CD8+ T cells increase in mild COPD cores was confirmed by CD8 immunostaining of the corresponding tissue cores (COPD, mild cores *vs* controls, log_2_FC = 2.37, p<0.001). CD8 immunostainings were also increased in moderate and severe COPD core groups as compared with controls, although to a lower extent (COPD, moderate cores *vs* controls, log_2_FC = 1.84, p<0.001; COPD, severe cores *vs* controls, log_2_FC = 1.07, p=0.01) (**Figures 4B, C**). Immunostaining for CD68 was increased in moderate COPD cores versus control cores (log_2_FC = 1.09, p=0.007), whereas CD4 and CD20 were also increased in moderate COPD cores (CD4: log_2_FC=2.57, p<0.001; CD20: log_2_FC=0.68 p=0.02) and in severe COPD cores *vs* control cores (CD4: log_2_FC=2.91, p<0.001; CD20: log_2_FC=0.80, p=0.01). An increase of collagen I (COL I) was also observed in moderate (log_2_FC = 0.85, p=0.05) and severe (log_2_FC = 0.98, p=0.03) COPD cores groups compared to controls. Immunostaining data for CD68, CD20, CD4 and COL I were unaffected in mild COPD cores group (**Figures 4B, C**).

## 4 Discussion

This study shows that infiltration of antigen presenting cells, CD4+ T cells, and B cells, along increased MHC II genes, occur within advanced emphysema zones of the COPD lung, while innate mucosal immunity alterations, including mucus and IgA production, are found in zones globally preserved from emphysematous destruction. Our study is the first that explores the interconnection between epithelial defense alterations and immune responses during COPD progression, by comparing zones with different tissue destruction levels, and suggests that impaired mucosal defenses could drive the disease and are not only a consequence of airway epithelial injury in the course of the disease.

First, a substantial decrease in several epithelial-derived defensins was observed already in early COPD zones. Previous studies reported increased airway levels of β-defensin 1 in COPD (23), whereas β-defensins 2 levels were decreased in COPD lung (24, 25). This decrease was, in these previous studies, only observed in tissue of active smokers and sputum of end-stage COPD lung. The decrease of several subtypes of defensins selectively in mild COPD zones in this study implies a possible causal role of this impairment in the course of the disease. As one study suggested a connection between β-defensins 2 deficiency and the inflammatory cells influx in the lung using an allergic asthma mouse model (26), a decreased production of some defensins could represent an early feature of mucosal defense dysregulation facilitating pathogens colonization and driving immune cells infiltration also in COPD.

Second, in those rather preserved zones from COPD lungs, mucin production was upregulated, along the transcription factor SPDEF promoting goblet cell differentiation. While several studies showed this cardinal feature of at all stages in chronic obstructive airway disease (27–29), our data are consistent with the SPIROMICS cohort study suggesting that MUC5AC overproduction may contribute to the initiation of COPD (30). In addition, it also infers that mucin overproduction occurs before adaptive immune response, suggesting a major role of mucociliary clearance alterations in driving the disease as well as the relevance of quantifying mucus plugs to assess the severity of chronic airway disease (31, 32).

In contrast with defensins, we identified in the same (mild) zones an upregulation of SLPI expression, in agreement with a previous study in sputum of smokers (33) and possibly following microbial-derived transcriptional regulation (34, 35), emphasizing the importance of airways pathogen colonization early in COPD.

The last key observation in mild zones from COPD lungs related to the activation of pathways involved in IgA production that is consistent with previous findings showing increased IgA+ B cells in COPD in end-stage COPD lung (13) and indicating that this signal is observed across all ranges from mild to severe tissue emphysema. It is tempting to speculate that increased IgA is initially produced in COPD lungs through innate mechanisms, while progression may involve adaptive pathways related to cognate interactions with (neo, self, microbial) antigens. In parallel, an upregulation of IL17 signaling pathway in mild, moderate and severe zones from COPD lungs is found in this study, confirming previous observations (36) supporting that IL17 plays a role in driving both innate and adaptive immune responses in COPD, and could represent a future therapeutic target (37).

In contrast with early disease zones, adaptive immune cells were increased in zones with advanced tissue destruction. However, CD8+ T cells and MHCI gene were increased at all levels of tissue destruction including -and even more particularly- in mild zones. The increase in CD8+ T cells and their cytotoxic potential are well described at all stages in COPD (12, 38, 39), possibly following a direct response to cigarette smoke exposure (40). Our findings suggest that CD8+ T cells preferentially increase at earlier stages of tissue destruction when comparing to the other adaptive immune cells, further emphasizing the need to investigate the role of CD8+ T cells in disease development.

Previous studies (28, 41) already showed a progressive increase of adaptive immune cells and connective tissue in COPD according to GOLD stages and emphysematous destruction, respectively. This study further shows that adaptive immunity is activated in the later stages of the disease, a concept supported by the observed upregulation in MHCII genes. Moreover, although a few studies have assessed the relationship between HLA alleles and COPD, this study is the first to quantify MHC genes in COPD lung tissue and its correlate with emphysematous destruction (42). In addition, a Th17 signature was also observed in severe COPD zones as previously reported (43). This increase in Th cells, as well as the absence of increase of neutrophils signature genes, confirms that emphysematous destruction is linked to an adaptive immune response and not only an innate immune response, as previously shown by others (41, 44). This study also shows that the increase in adaptive immune cells in zones with advanced emphysema goes along an increased collagen deposition, as previously described (45). These data further reinforce suggestions that the extracellular matrix turnover is increased in COPD (46), and that components of the matrix could be used a biomarkers of airway remodelling (47).

This study also confirms that terminal bronchioles are already decreased in mild COPD zones (4, 48), further indicating that tissue destruction affects both the alveoli and the terminal bronchioles and that small airways disappearance is an early feature of the disease (4). This study also shows that this decrease in the number of terminal bronchioles starts before the onset of adaptive immune responses. An other study using cores from diversely affected zones of COPD explant lungs found a Th and B cells infiltration in zones of micro-emphysema, determined by a mean linear intercept measurement, where terminal bronchioles decreased (49). Thresholding of the zones of mild, moderate and severe emphysema can explain the discrepancy between the onset of the adaptive immune response, the degree of emphysema and the decrease of terminal bronchioles. This study is, however, consistent with the fact that there is decrease in terminal bronchioles and an increase in adaptive immune cell infiltration with increasing emphysema in COPD explant lungs.

Finally, we identified FAM46C as a top differentially expressed gene in our COPD cores as compared with controls, as described in previous studies (50, 51); its role which is globally unknown should be addressed in future studies.

This study has several limitations. First, while the approach enabled to relate molecular and cellular data to tissue structural changes, it remains uncertain whether those changes occurring in mild *vs* severe zones actually reflect early *vs* late stages of the diseases; alternatively, those zones could reflect a pure spatial heterogeneity. A second limitation relates to the fact that the RNAseq method fails to sequence immunoglobulin genes, and therefore did not enable to directly assess IgA expression in the different.

samples. This should prompt further studies to address this point.

**Figure 5:**
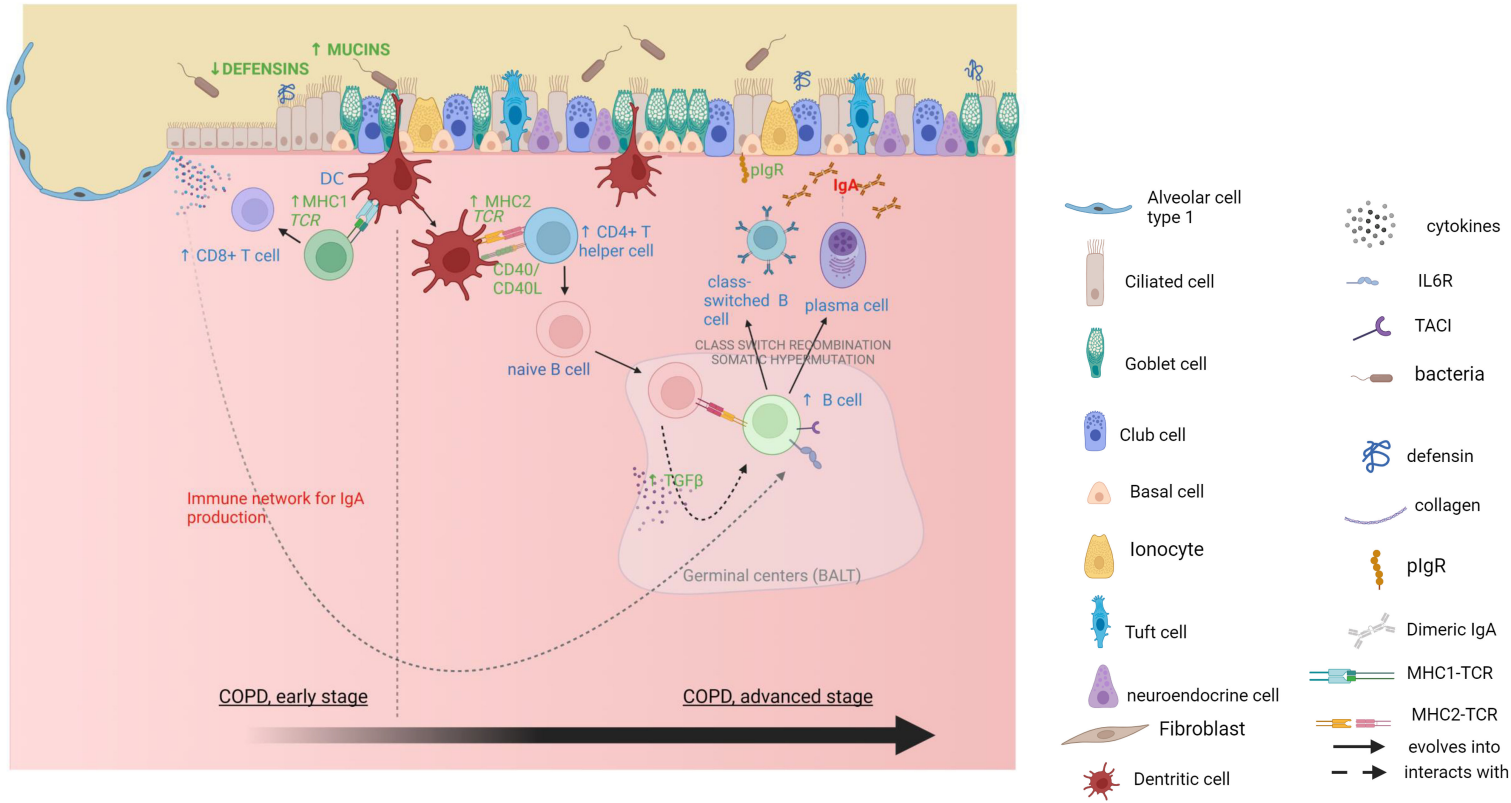
Mucosal immunity alterations and CD8+ T cells activation in mild COPD cores, followed by an adaptive IgA response in moderate and severe COPD cores. Gene expression data are in *green*, xCell deconvolution data are in *blue*, pathway enrichment data are in *red*. Created with *BioRender.com* .

Overall, this study indicates that during COPD development early alterations in mucosal defense could drive a failure of the epithelial barrier against inhaled toxics and pathogens, which are in turn progressively promoting adaptive immune responses (**Figure 5**), leading to a vicious circle of altered frontline defense and dysregulated adaptive immunity ultimately underlying the tissue destruction in COPD. Future studies should focus on the causal relationship between immunity and tissue destruction as well as on barrier defense changes occurring at early stages, before irreversible lesions establish.

## Data availability statement

The datasets presented in this study can be found in online repositories. The names of the repository/repositories and accession number(s) can be found below: https://www.ncbi.nlm.nih.gov/geo/, GSE239897.

## Ethics statement

The studies involving humans were approved by Medical Ethics Board of University Hospitals Leuven, Belgium (S52174). The studies were conducted in accordance with the local legislation and institutional requirements. The participants provided their written informed consent to participate in this study.

## Author contributions

CdF: Formal Analysis, Investigation, Visualization, Writing – original draft, Writing – review & editing. VG: Investigation, Visualization, Writing – original draft, Writing – review & editing. IG: Investigation, Visualization, Writing – original draft, Writing – review & editing. PK: Visualization, Writing – original draft, Writing – review & editing, Investigation. AV: Investigation, Visualization, Writing – original draft, Writing – review & editing. TG: Investigation, Visualization, Writing – original draft, Writing – review & editing. MV: Investigation, Visualization, Writing – original draft, Writing – review & editing. HB: Investigation, Visualization, Writing – original draft, Writing – review & editing. JK: Investigation, Visualization, Writing – original draft, Writing – review & editing. JV: Investigation, Visualization, Writing – original draft, Writing – review & editing. YM: Investigation, Visualization, Writing – original draft, Writing – review & editing. LW: Investigation, Visualization, Writing – original draft, Writing – review & editing. LA: Investigation, Visualization, Writing – original draft, Writing – review & editing. EC: Investigation, Visualization, Writing – original draft, Writing – review & editing. CH: Investigation, Visualization, Writing – original draft, Writing – review & editing. GA: Investigation, Visualization, Writing – original draft, Writing – review & editing. CA: Investigation, Visualization, Writing – original draft, Writing – review & editing. SE: Writing – original draft, Writing – review & editing. JM: Investigation, Methodology, Visualization, Writing – original draft, Writing – review & editing. LD: Investigation, Methodology, Visualization, Writing – original draft, Writing – review & editing. SG: Writing – original draft, Writing – review & editing. JA: Formal Analysis, Software, Validation, Visualization, Writing – original draft, Writing – review & editing. WJ: Writing – original draft, Writing – review & editing. LC: Writing – original draft, Writing – review & editing. DV: Writing – original draft, Writing – review & editing. RV: Writing – original draft, Writing – review & editing. TH: Methodology, Writing – original draft, Writing – review & editing. JH: Methodology, Writing – original draft, Writing – review & editing. NK: Methodology, Writing – original draft, Writing – review & editing. GG-R: Visualization, Writing – original draft, Writing – review & editing. CP: Supervision, Visualization, Writing – original draft, Writing – review & editing. BV: Conceptualization, Methodology, Supervision, Visualization, Writing – original draft, Writing – review & editing.
