# Supplementary material for: Mucosal immune alterations at the early onset of tissue destruction in chronic obstructive pulmonary disease
Source: Front Immunol. 2023 Oct 17;14:1275845. doi: 10.3389/fimmu.2023.1275845 (PMC10616299; doi:10.3389/fimmu.2023.1275845)
Supplement: Supplementary file 1 [file DataSheet_1.docx]

Supplementary Material

Mucosal immune alterations at the early onset of tissue destruction in Chronic Obstructive Pulmonary Disease

Charlotte de Fays^1,2^, Vincent Geudens^2^, Iwein Gyselinck^2^, Pieterjan Kerckhof^2^, Astrid Vermaut^2^, Tinne Goos^2^, Marie Vermant^2^, Hanne Beeckmans^2^, Janne Kaes^2^, Jan Van Slambrouck^2^, Yousry Mohamady^2^, Lynn Willems^2^, Lucia Aversa^2^, Emanuela E. Cortesi^2^, Charlotte Hooft^2^, Gitte Aerts^2^, Celine Aelbrecht^2^, Stephanie Everaerts^2^, John E. Mcdonough^4^, Laurens J. De Sadeleer^2^, Sophie Gohy^1,3^, Jerome Ambroise^5^, Wim Janssens^2^, Laurens J. Ceulemans^2^, Dirk Van Raemdonck^2^, Robin Vos^2^, Tillie L. Hackett^6^, James C. Hogg^6^, Naftali Kaminski^4^, Ghislaine Gayan-Ramirez^2^, Charles Pilette^1,3†^, Bart M. Vanaudenaerde^2†^**^*^**

^†^ These authors contributed equally to this work and share last authorship

- ^1^Pole of Pneumology, ENT, and Dermatology, Institute of Experimental and Clinical Research, Université Catholique de Louvain, Brussels, Belgium,

^2^Laboratory of Respiratory Diseases and Thoracic Surgery, BREATHE, Department of CHROMETA, KU Leuven, Leuven, Belgium,

^3^Department of pneumology, Cliniques Universitaires Saint-Luc, Brussels, Belgium

^4^Section of Pulmonary, Critical Care, and Sleep Medicine, Yale University School of Medicine, New Haven, CT, United States,

^5^Centre de Technologies Moléculaires Appliquées, Institute of Experimental and Clinical Research, Université Catholique de Louvain, Brussels, Belgium,

^6^Centre for Heart Lung Innovation, St Paul's Hospital, Vancouver, B.C., BC, Canada.

***Correspondence:**

Prof. Dr. Bart Vanaudenaerde

Department of Chronic Diseases, and Metabolism (CHROMETA),

Laboratory of Respiratory Diseases and Thoracic Surgery (BREATHE),

KU Leuven and UZ Leuven; Herestraat 49, B-3000 Leuven, Belgium

Tel: + 32 16 330194 Fax: + 32 16 346803 E-mail: [bart.vanaudenaerde@kuleuven.be](mailto:bart.vanaudenaerde@kuleuven.be)

# Supplementary Data

**Table E1** : Top differentially expressed genes analysis

COPD, mild cores vs controls

| Genes | logFC | logCPM | F | PValue | FDR |
| --- | --- | --- | --- | --- | --- |
| HBA2 | 5.42 | 10.9 | 104 | 4.93E-16 | 4.24E-12 |
| HBA1 | 5.42 | 10.9 | 104 | 4.93E-16 | 4.24E-12 |
| TRIB1 | 2.26 | 6.77 | 88.7 | 1.53E-14 | 8.76E-11 |
| HBB | 4.74 | 9.96 | 79.5 | 1.44E-13 | 6.2E-10 |
| PTGS2 | 2.49 | 7.16 | 69 | 2.23E-12 | 7.68E-09 |
| CYR61 | 1.99 | 9.27 | 49.9 | 5.62E-10 | 1.61E-06 |
| ODF3L1 | 3.02 | 0.369 | 49.3 | 6.84E-10 | 1.68E-06 |
| JUND | 1.39 | 7.49 | 43.2 | 4.93E-09 | 1.06E-05 |
| ADCYAP1 | 4.44 | 0.259 | 40.4 | 1.27E-08 | 2.42E-05 |
| EGR3 | 2.71 | 4.57 | 39.3 | 1.83E-08 | 2.91E-05 |
| ALAS2 | 3.73 | 0.83 | 39.2 | 1.88E-08 | 2.91E-05 |
| RASGEF1B | 1.16 | 6.48 | 38.8 | 2.18E-08 | 2.91E-05 |
| VPS37B | 0.987 | 6.79 | 38.7 | 2.2E-08 | 2.91E-05 |
| HBG1 | 5.32 | 5.02 | 37.2 | 3.78E-08 | 4.25E-05 |
| HBG2 | 5.32 | 5.02 | 37.2 | 3.8E-08 | 4.25E-05 |
| RND3 | 1.09 | 6.12 | 37 | 3.95E-08 | 4.25E-05 |
| FAM46C | 1.19 | 5.85 | 36.7 | 4.39E-08 | 0.000044 |
| MBNL1-AS1 | 0.864 | 3.81 | 36.6 | 4.6E-08 | 0.000044 |
| MYADM | 1.4 | 7.8 | 34.6 | 9.37E-08 | 8.48E-05 |
| NR4A3 | 2.63 | 5.35 | 34.2 | 1.08E-07 | 9.31E-05 |
| SNCA | 1.07 | 3.91 | 33.6 | 1.35E-07 | 0.000109 |
| KLF10 | 1.42 | 6.61 | 33.4 | 1.42E-07 | 0.000109 |
| DYRK3 | 1.44 | 4.39 | 33.4 | 1.45E-07 | 0.000109 |
| B3GNT5 | 1.28 | 5.32 | 32.8 | 1.77E-07 | 0.000127 |
| TRIL | 1.77 | 3.08 | 32.6 | 1.92E-07 | 0.000132 |
| MPZL3 | 1.08 | 4.93 | 32.4 | 2.03E-07 | 0.000135 |
| CTGF | 1.66 | 7.94 | 31.8 | 2.61E-07 | 0.000166 |
| OTUD1 | 1.01 | 5.49 | 31.2 | 3.21E-07 | 0.000197 |
| BTG2 | 1.68 | 8.08 | 30.8 | 3.77E-07 | 0.000217 |
| HOMER1 | 0.935 | 5.18 | 30.7 | 3.78E-07 | 0.000217 |
| HBEGF | 1.59 | 7.63 | 30.2 | 4.58E-07 | 0.000254 |
| HBD | 2.79 | 2.48 | 30 | 5.07E-07 | 0.000273 |
| STEAP4 | 1.36 | 7.42 | 29.7 | 5.52E-07 | 0.000288 |
| LDLR | 1.18 | 8.53 | 29 | 7.26E-07 | 0.000367 |
| SERPIND1 | 2.81 | 2.9 | 28.8 | 7.79E-07 | 0.000373 |
| MSANTD4 | -0.644 | 4.08 | 28.8 | 7.81E-07 | 0.000373 |
| DUSP5 | 1.59 | 4.41 | 28.6 | 8.34E-07 | 0.000388 |
| ZFP36L1 | 0.772 | 7.95 | 28.3 | 9.37E-07 | 0.000424 |
| ZNF785 | -0.887 | 3.73 | 28.1 | 0.000001 | 0.000443 |
| SRF | 0.695 | 5.65 | 27.8 | 1.14E-06 | 0.000492 |
| SOCS2 | 2.64 | 4.64 | 27.7 | 1.18E-06 | 0.000497 |
| VMP1 | 1.02 | 8.4 | 27.2 | 1.46E-06 | 0.000592 |
| C10orf67 | 1.75 | 4.59 | 27.1 | 1.48E-06 | 0.000592 |
| YPEL5 | 0.607 | 8.02 | 27 | 1.57E-06 | 0.000606 |
| PRKCQ-AS1 | 1.22 | 3.31 | 27 | 1.59E-06 | 0.000606 |
| SLMO1 | -1.08 | 2.89 | 26.9 | 1.62E-06 | 0.000606 |
| GPR183 | 1.82 | 4.99 | 26.5 | 1.87E-06 | 0.000686 |
| BLID | 1.41 | 2.32 | 26 | 2.29E-06 | 0.000821 |
| PTPN18 | -0.551 | 5.17 | 25.9 | 2.41E-06 | 0.000845 |
| KRT17 | 3.11 | 5.8 | 25.7 | 2.57E-06 | 0.000883 |
| TP53INP2 | 1.12 | 3.66 | 25.5 | 2.78E-06 | 0.000938 |
| FAM213B | -0.664 | 4.63 | 25.5 | 2.84E-06 | 0.000939 |
| CYSTM1 | 0.57 | 6.45 | 25.2 | 3.15E-06 | 0.00102 |
| PPP1R10 | 0.888 | 7.28 | 25.1 | 3.3E-06 | 0.00105 |
| PTP4A1 | 0.932 | 7.21 | 25 | 3.45E-06 | 0.00107 |
| C11orf96 | 1.5 | 6.75 | 24.9 | 3.49E-06 | 0.00107 |
| INHBA | 1.42 | 5.94 | 24.8 | 3.69E-06 | 0.00111 |
| MED8 | -0.529 | 5.14 | 24.7 | 3.81E-06 | 0.00113 |
| ZNF451 | 0.519 | 4.64 | 24.6 | 3.93E-06 | 0.00115 |
| NAIP | -1.28 | 3.95 | 24.5 | 4.06E-06 | 0.00116 |
| WEE1 | 1.27 | 5.6 | 24.5 | 4.16E-06 | 0.00117 |
| JMJD1C | 0.866 | 6.74 | 24.2 | 4.63E-06 | 0.00128 |
| RRAD | 1.63 | 4.31 | 24.2 | 4.7E-06 | 0.00128 |
| SELE | 2.83 | 4.15 | 24 | 5.07E-06 | 0.00136 |
| SURF6 | -0.641 | 5.51 | 23.9 | 5.24E-06 | 0.00137 |
| MBNL1 | 0.465 | 5.03 | 23.9 | 5.25E-06 | 0.00137 |
| LOC100131496 | -1.38 | 3.21 | 23.9 | 5.34E-06 | 0.00137 |
| CHFR | -0.565 | 5.22 | 23.8 | 5.43E-06 | 0.00137 |
| GADD45B | 1.39 | 7.39 | 23.8 | 5.57E-06 | 0.00139 |
| CD69 | 1.54 | 6.3 | 23.6 | 5.83E-06 | 0.00142 |
| BHLHE40 | 1.44 | 8.67 | 23.6 | 5.87E-06 | 0.00142 |
| DUSP1 | 1.45 | 11 | 23.5 | 6.15E-06 | 0.00147 |
| DNAJB4 | 0.862 | 6.51 | 23.4 | 6.4E-06 | 0.00148 |
| SPTY2D1 | 0.527 | 5.32 | 23.4 | 6.43E-06 | 0.00148 |
| SMARCD3 | -0.722 | 4.38 | 23.4 | 6.5E-06 | 0.00148 |
| TOPBP1 | -0.421 | 5.11 | 23.3 | 6.54E-06 | 0.00148 |
| IL20RA | 1.53 | 3.48 | 23.3 | 6.62E-06 | 0.00148 |
| CHRNA1 | 3.64 | 1.54 | 23.2 | 6.98E-06 | 0.00153 |
| TXLNB | 1.63 | 2.45 | 23.2 | 7.01E-06 | 0.00153 |
| EGR2 | 1.79 | 6.08 | 23 | 7.53E-06 | 0.00162 |
| MAML2 | 0.874 | 5.47 | 23 | 7.62E-06 | 0.00162 |
| BDKRB2 | 2.12 | 1.2 | 22.9 | 7.95E-06 | 0.00167 |
| KLF5 | 0.923 | 6.63 | 22.6 | 8.98E-06 | 0.00186 |
| MED26 | 0.85 | 2.64 | 22.5 | 9.32E-06 | 0.00188 |
| LMNA | 1.39 | 8.52 | 22.4 | 9.4E-06 | 0.00188 |
| GCFC2 | 0.576 | 3.39 | 22.4 | 9.56E-06 | 0.00188 |
| WDR5 | -0.406 | 4.66 | 22.4 | 9.59E-06 | 0.00188 |
| MGAT4B | -0.424 | 6.49 | 22.4 | 9.6E-06 | 0.00188 |
| CTSV | 1.89 | 1.86 | 22.2 | 1.02E-05 | 0.00196 |
| RTCA | -0.566 | 5.63 | 22.2 | 1.03E-05 | 0.00196 |
| RNF122 | 1.17 | 5.71 | 22.2 | 1.04E-05 | 0.00197 |
| CCDC142 | -0.805 | 4.56 | 22.1 | 1.09E-05 | 0.00203 |
| NPM3 | -0.96 | 3.84 | 22.1 | 0.000011 | 0.00204 |
| SEMA3E | 1.32 | 3.04 | 22 | 1.12E-05 | 0.00205 |
| HLA-G | 3.33 | 5.67 | 21.9 | 1.19E-05 | 0.00216 |
| VIPAS39 | -0.489 | 3.79 | 21.8 | 1.23E-05 | 0.0022 |
| JUNB | 1.55 | 9.01 | 21.7 | 1.29E-05 | 0.0023 |
| ATXN7 | 1.19 | 2.54 | 21.6 | 1.32E-05 | 0.00232 |
| CLN3 | -0.451 | 5.61 | 21.4 | 1.44E-05 | 0.00251 |
| PPP1R15A | 1.29 | 5.82 | 21.2 | 1.55E-05 | 0.00267 |
| TCP11L2 | 0.663 | 5.78 | 21.2 | 1.58E-05 | 0.00267 |
| MLF1 | 1.4 | 5.43 | 21.2 | 1.58E-05 | 0.00267 |
| ZFP36L2 | 0.636 | 7.83 | 21.1 | 1.61E-05 | 0.0027 |
| SLC4A1 | 3.08 | 0.785 | 21.1 | 1.65E-05 | 0.00272 |
| CTC-338M12.4 | 1.39 | 1.15 | 20.9 | 1.74E-05 | 0.00285 |
| RBM45 | -0.903 | 4.05 | 20.9 | 1.78E-05 | 0.00288 |
| ABO | 1.29 | 2.75 | 20.9 | 1.79E-05 | 0.00288 |
| MYO1E | 0.699 | 6.01 | 20.8 | 1.87E-05 | 0.00297 |
| ANXA1 | 0.826 | 9.57 | 20.8 | 1.88E-05 | 0.00297 |
| KLF11 | 0.675 | 6.16 | 20.7 | 1.91E-05 | 0.00299 |
| ATF3 | 2.27 | 7.05 | 20.7 | 1.93E-05 | 0.003 |
| FCMR | 1.09 | 3.53 | 20.6 | 1.97E-05 | 0.00303 |
| AMZ2P1 | 0.923 | 2.57 | 20.6 | 2.02E-05 | 0.00307 |
| KRT16P1 | 3.03 | 3.27 | 20.5 | 2.06E-05 | 0.00309 |
| HNRNPLL | 0.47 | 5.61 | 20.5 | 2.07E-05 | 0.00309 |
| AGR3 | 1.38 | 7.27 | 20.4 | 2.19E-05 | 0.00325 |
| WSB1 | 0.972 | 6.55 | 20.3 | 2.23E-05 | 0.00326 |
| DUSP10 | 0.734 | 4.7 | 20.3 | 2.24E-05 | 0.00326 |
| TRIM52 | 1.29 | 1.28 | 20.2 | 2.33E-05 | 0.00336 |
| RARRES3 | -0.696 | 7.72 | 20.1 | 2.45E-05 | 0.00351 |
| DUSP12 | -0.516 | 4.18 | 20 | 2.52E-05 | 0.00359 |
| LINC00312 | 1.36 | 4.44 | 20 | 0.000026 | 0.00367 |
| ZFP36 | 1.79 | 9.7 | 19.9 | 2.64E-05 | 0.00369 |
| EGR1 | 2.16 | 8.57 | 19.9 | 2.67E-05 | 0.0037 |
| FOSB | 3.19 | 8.89 | 19.9 | 2.69E-05 | 0.0037 |
| CD9 | 0.565 | 9.11 | 19.8 | 0.000028 | 0.00383 |
| SMAD3 | 0.456 | 5.82 | 19.6 | 2.99E-05 | 0.00401 |
| GJA1 | 0.882 | 7.57 | 19.6 | 2.99E-05 | 0.00401 |
| COL10A1 | 2.38 | 0.814 | 19.6 | 0.00003 | 0.00401 |
| SPATA20 | -0.993 | 3.86 | 19.6 | 3.08E-05 | 0.00408 |
| CDKN1A | 1.23 | 6.89 | 19.5 | 3.19E-05 | 0.00418 |
| TACSTD2 | 1.06 | 7.33 | 19.4 | 3.29E-05 | 0.00428 |
| RORB | -1.27 | 2.4 | 19.4 | 3.31E-05 | 0.00428 |
| C2CD4B | 1.87 | 3.61 | 19.3 | 3.39E-05 | 0.00435 |
| POLH | -0.519 | 4.8 | 19.3 | 3.44E-05 | 0.00438 |
| TARSL2 | 0.474 | 5 | 19.2 | 3.57E-05 | 0.00451 |
| SLC12A9 | -0.517 | 4.92 | 19.2 | 3.59E-05 | 0.00451 |
| HSPA7 | -1.28 | 5.15 | 19.1 | 3.69E-05 | 0.00459 |
| DOCK4 | 0.603 | 5.65 | 19.1 | 3.73E-05 | 0.00459 |
| CXADR | 0.72 | 5.95 | 19.1 | 3.74E-05 | 0.00459 |
| PSTPIP1 | -0.99 | 3.13 | 19.1 | 3.81E-05 | 0.00465 |
| RPS15AP10 | -0.821 | 3.44 | 19 | 3.87E-05 | 0.00469 |
| TIMM13 | -0.592 | 4.32 | 18.9 | 4.05E-05 | 0.00487 |
| APLN | 1.83 | 2.75 | 18.9 | 4.07E-05 | 0.00487 |
| TIGD1 | -0.805 | 3.71 | 18.8 | 0.000042 | 0.00499 |
| TTL | -0.53 | 5.31 | 18.8 | 4.34E-05 | 0.00511 |
| SFTA1P | 1.04 | 7.2 | 18.7 | 4.41E-05 | 0.00516 |
| SLC41A1 | 0.488 | 4.86 | 18.6 | 4.55E-05 | 0.00529 |
| ARL5B | 0.773 | 5.42 | 18.6 | 4.69E-05 | 0.00542 |
| BBIP1 | 0.571 | 4.36 | 18.5 | 4.75E-05 | 0.00544 |
| FOXA2 | 0.828 | 2.01 | 18.5 | 4.77E-05 | 0.00544 |
| NR4A1 | 2.1 | 6.46 | 18.5 | 4.81E-05 | 0.00544 |
| KLHL15 | 0.637 | 3.11 | 18.5 | 4.84E-05 | 0.00544 |
| SLC25A33 | 0.799 | 3.32 | 18.5 | 4.91E-05 | 0.00545 |
| CENPQ | 0.611 | 3.49 | 18.5 | 4.91E-05 | 0.00545 |
| CFD | 0.867 | 6.79 | 18.4 | 4.94E-05 | 0.00545 |
| NHSL1 | 0.741 | 4.51 | 18.4 | 4.98E-05 | 0.00545 |
| CACNB1 | -0.926 | 4.03 | 18.4 | 5.02E-05 | 0.00545 |
| ZKSCAN4 | -0.831 | 2.25 | 18.4 | 5.04E-05 | 0.00545 |
| PELI1 | 1.01 | 6.87 | 18.4 | 0.000051 | 0.00549 |
| CDC25B | -0.89 | 6.49 | 18.3 | 5.36E-05 | 0.00573 |
| TBC1D24 | -0.799 | 5.26 | 18.2 | 5.39E-05 | 0.00573 |
| EDRF1 | -0.661 | 4.08 | 18.1 | 5.69E-05 | 0.00601 |
| SERTAD1 | 1.19 | 4.71 | 18.1 | 5.75E-05 | 0.00603 |
| EPHA2 | 1.11 | 6.25 | 18 | 5.88E-05 | 0.00613 |
| SLC24A4 | -1.02 | 2.11 | 18 | 5.95E-05 | 0.00616 |
| NR4A2 | 1.92 | 5.24 | 17.9 | 6.19E-05 | 0.00638 |
| PSTPIP2 | -0.749 | 4.05 | 17.9 | 6.25E-05 | 0.0064 |
| ZSCAN20 | -0.843 | 1.81 | 17.8 | 6.54E-05 | 0.00663 |
| TP53I3 | -0.888 | 4.27 | 17.8 | 6.56E-05 | 0.00663 |
| ZNF707 | -0.576 | 4.17 | 17.8 | 6.64E-05 | 0.00668 |
| PRR15L | -1.08 | 5.54 | 17.7 | 6.79E-05 | 0.00678 |
| ERBB3 | 0.673 | 6.08 | 17.7 | 6.82E-05 | 0.00678 |
| LPIN1 | -0.511 | 5.05 | 17.7 | 6.85E-05 | 0.00678 |
| P2RY1 | -0.911 | 4.78 | 17.7 | 0.000069 | 0.00678 |
| PARD6B | 0.666 | 5.68 | 17.6 | 0.00007 | 0.00684 |
| SARAF | 0.374 | 8.4 | 17.6 | 7.24E-05 | 0.00701 |
| SOCS3 | 1.8 | 5.93 | 17.5 | 7.27E-05 | 0.00701 |
| TATDN1 | 0.683 | 5.27 | 17.5 | 0.000073 | 0.00701 |
| SMG9 | -0.43 | 4.77 | 17.5 | 7.39E-05 | 0.00706 |
| CIT | 1.44 | 4.26 | 17.4 | 0.000076 | 0.00722 |
| BRMS1 | -0.721 | 4.47 | 17.4 | 7.71E-05 | 0.00728 |
| MAD1L1 | -0.415 | 5.32 | 17.3 | 7.92E-05 | 0.00742 |
| C15orf57 | 0.415 | 5.24 | 17.3 | 7.94E-05 | 0.00742 |
| POLM | -0.917 | 3.13 | 17.3 | 8.03E-05 | 0.00747 |
| TUBA1A | 0.873 | 8.66 | 17.3 | 8.09E-05 | 0.00748 |
| RBM12B-AS1 | -0.86 | 4.36 | 17.3 | 0.000082 | 0.00755 |
| SERPINB5 | 3.86 | 1.93 | 17.2 | 8.37E-05 | 0.00766 |
| AHSP | 2.88 | -0.348 | 17.2 | 8.54E-05 | 0.00774 |
| LGALS9C | 2.63 | 1.78 | 17.2 | 8.54E-05 | 0.00774 |
| AMDHD2 | -0.544 | 4.62 | 17.1 | 8.67E-05 | 0.00781 |
| SUN1 | 0.552 | 7.67 | 17.1 | 8.83E-05 | 0.00791 |
| BMP2 | 1.28 | 5.41 | 17 | 9.13E-05 | 0.00814 |
| SH3BP2 | -0.881 | 3.07 | 17 | 9.26E-05 | 0.00817 |
| MIDN | 0.921 | 5.21 | 17 | 9.33E-05 | 0.00817 |
| CSF3 | 2.65 | 4.4 | 17 | 9.34E-05 | 0.00817 |
| ZNF101 | -0.598 | 3.62 | 16.9 | 0.000094 | 0.00817 |
| MUC13 | 3.82 | 2.36 | 16.9 | 9.41E-05 | 0.00817 |
| TES | 0.488 | 6.49 | 16.9 | 9.58E-05 | 0.00823 |
| SDHAF1 | -0.855 | 3.44 | 16.9 | 9.61E-05 | 0.00823 |
| NOB1 | -0.607 | 4.7 | 16.9 | 9.69E-05 | 0.00823 |
| SLC25A38 | -0.511 | 4.46 | 16.9 | 9.75E-05 | 0.00823 |
| MET | 0.766 | 6.57 | 16.9 | 9.75E-05 | 0.00823 |
| CNTF | 2.66 | 1.11 | 16.9 | 9.76E-05 | 0.00823 |
| DUSP2 | 1.43 | 2.75 | 16.8 | 9.88E-05 | 0.00829 |
| KRT6B | 3.31 | 0.563 | 16.8 | 0.000102 | 0.00847 |
| BSDC1 | 0.386 | 5.75 | 16.7 | 0.000103 | 0.00847 |
| IRF6 | 1.03 | 4.6 | 16.7 | 0.000103 | 0.00847 |
| CDC123 | -0.314 | 6.49 | 16.7 | 0.000103 | 0.00847 |
| TC2N | 0.796 | 6.48 | 16.7 | 0.000103 | 0.00847 |
| IDS | 0.398 | 7.4 | 16.7 | 0.000105 | 0.00856 |
| RABGGTB | -0.427 | 6.28 | 16.7 | 0.000106 | 0.00858 |
| POGLUT1 | -0.528 | 4.46 | 16.5 | 0.000112 | 0.009 |
| UAP1L1 | -0.923 | 3.61 | 16.5 | 0.000112 | 0.009 |
| GALC | 0.482 | 6.19 | 16.5 | 0.000113 | 0.00902 |
| TSPYL1 | 0.332 | 7.14 | 16.5 | 0.000113 | 0.009 |
| CAD | -0.571 | 3.87 | 16.5 | 0.000114 | 0.00908 |
| C1GALT1 | 0.604 | 5.61 | 16.4 | 0.000118 | 0.0093 |
| TELO2 | -0.52 | 4.35 | 16.4 | 0.000121 | 0.00944 |
| ARGFXP2 | -0.84 | 3.65 | 16.4 | 0.000121 | 0.00944 |
| SPDL1 | -0.598 | 3.65 | 16.4 | 0.000121 | 0.00944 |
| SERTAD3 | 0.607 | 4.69 | 16.3 | 0.000123 | 0.00948 |
| C1QTNF7 | 1.41 | 2.78 | 16.3 | 0.000123 | 0.00948 |
| FBXW5 | -0.346 | 5.95 | 16.3 | 0.000123 | 0.00948 |
| BAX | -0.623 | 5.85 | 16.3 | 0.000125 | 0.00955 |
| RHBDD3 | -0.89 | 3.87 | 16.3 | 0.000125 | 0.00955 |
| SLC35G6 | -1.71 | 1.66 | 16.2 | 0.000128 | 0.00968 |
| RARRES2 | 0.709 | 7.41 | 16.2 | 0.000132 | 0.00994 |
| AHR | 0.642 | 6.72 | 16.2 | 0.000133 | 0.00997 |
| CFAP20 | 0.627 | 4.22 | 16.1 | 0.000134 | 0.01 |
| SGK2 | -1.15 | 1.19 | 16.1 | 0.000134 | 0.01 |
| RPS19BP1 | -0.506 | 4.68 | 16.1 | 0.000135 | 0.01 |
| MCTS2P | -0.612 | 4.52 | 16.1 | 0.000137 | 0.0101 |
| WBSCR27 | 2.04 | 1.02 | 16.1 | 0.000137 | 0.0101 |
| TMEM255A | 1.88 | 1.58 | 16.1 | 0.000138 | 0.0101 |
| USP35 | -0.602 | 3.41 | 16 | 0.00014 | 0.0102 |
| SAYSD1 | -0.609 | 2.41 | 16 | 0.000143 | 0.0103 |
| MUC15 | 1.4 | 4.02 | 16 | 0.000144 | 0.0104 |
| GRAMD3 | 0.89 | 4.2 | 16 | 0.000144 | 0.0104 |
| MAPK6 | 0.564 | 5.9 | 15.9 | 0.000146 | 0.0104 |
| PSMC5 | -0.547 | 5.5 | 15.9 | 0.000146 | 0.0104 |
| ZBTB21 | 0.693 | 4.85 | 15.9 | 0.000147 | 0.0105 |
| EML3 | -0.553 | 4.72 | 15.9 | 0.000148 | 0.0105 |
| TRIM65 | -0.616 | 3.83 | 15.9 | 0.000149 | 0.0105 |
| PPP1R15B | 0.67 | 6.16 | 15.9 | 0.00015 | 0.0106 |
| NAF1 | 0.445 | 5.37 | 15.9 | 0.000151 | 0.0106 |
| TMEM106A | -0.711 | 4.32 | 15.8 | 0.000153 | 0.0106 |
| MYC | 1.35 | 5.43 | 15.8 | 0.000156 | 0.0108 |
| DUSP26 | 1.45 | 1.18 | 15.8 | 0.000157 | 0.0109 |
| SLC7A6OS | -0.437 | 4.73 | 15.7 | 0.00016 | 0.0109 |
| SNAPC3 | -0.422 | 6.52 | 15.7 | 0.00016 | 0.0109 |
| FAM65C | -1.57 | 2.41 | 15.7 | 0.000161 | 0.0109 |
| IL6 | 2.09 | 5.65 | 15.7 | 0.000161 | 0.0109 |
| ZSCAN12 | -0.629 | 3.74 | 15.7 | 0.000162 | 0.011 |
| CHMP1B | 0.336 | 7.1 | 15.7 | 0.000163 | 0.011 |
| CD83 | 1.16 | 5.39 | 15.6 | 0.000169 | 0.0114 |
| KRT6C | 3.06 | 0.143 | 15.6 | 0.000171 | 0.0114 |
| CRIP1 | 1.05 | 5.71 | 15.5 | 0.000173 | 0.0116 |
| RPS6KA4 | -0.664 | 4.3 | 15.5 | 0.000175 | 0.0116 |
| EMP1 | 1.23 | 8.23 | 15.5 | 0.000178 | 0.0118 |
| LOC440434 | -0.41 | 7.09 | 15.5 | 0.000179 | 0.0118 |
| KBTBD2 | 0.487 | 5.22 | 15.4 | 0.000184 | 0.0121 |
| TSC22D2 | 1.37 | 0.836 | 15.4 | 0.000185 | 0.0121 |
| HERC2P2 | -0.61 | 6.38 | 15.4 | 0.000188 | 0.0123 |
| UHRF1BP1 | -0.471 | 4.64 | 15.3 | 0.00019 | 0.0123 |
| NPEPPS | -0.302 | 7.94 | 15.3 | 0.000194 | 0.0125 |
| SHMT2 | -0.93 | 4.34 | 15.2 | 0.000201 | 0.0129 |
| OSGIN2 | 0.63 | 5.97 | 15.2 | 0.000201 | 0.0129 |
| RNF213 | -0.506 | 6.93 | 15.2 | 0.000205 | 0.0131 |
| IMPDH1 | -0.631 | 5.52 | 15.1 | 0.000207 | 0.0132 |
| TMEM30B | 0.625 | 6.64 | 15.1 | 0.000208 | 0.0132 |
| SNF8 | -0.453 | 5.2 | 15.1 | 0.000212 | 0.0133 |
| IP6K2 | 0.534 | 4.74 | 15.1 | 0.000212 | 0.0133 |
| NT5DC2 | -0.597 | 5.27 | 15.1 | 0.000212 | 0.0133 |
| ZC3H12A | 1.21 | 4.52 | 15.1 | 0.000214 | 0.0134 |
| SERPINB2 | 2.68 | 0.913 | 15.1 | 0.000216 | 0.0134 |
| KBTBD4 | 0.432 | 4.97 | 15 | 0.000218 | 0.0135 |
| LOC100129917 | -0.7 | 4.86 | 15 | 0.00022 | 0.0136 |
| BCL10 | 0.52 | 5.21 | 15 | 0.000221 | 0.0136 |
| TFAP2C | 0.867 | 3.66 | 15 | 0.000222 | 0.0136 |
| CXCL3 | 1.87 | 5.56 | 15 | 0.000222 | 0.0136 |
| EGF | -1.09 | 2.09 | 15 | 0.000223 | 0.0136 |
| MAGOH | 0.423 | 5.26 | 15 | 0.000224 | 0.0136 |
| SMG5 | -0.392 | 6.41 | 15 | 0.000225 | 0.0136 |
| ERRFI1 | 1.38 | 2.22 | 14.9 | 0.000226 | 0.0137 |
| FAH | -0.716 | 3.99 | 14.9 | 0.000231 | 0.0139 |
| DUX4 | 3.93 | 0.914 | 14.9 | 0.000232 | 0.0139 |
| FBN3 | 1.47 | 1.25 | 14.9 | 0.000233 | 0.0139 |
| CDON | 0.765 | 3.29 | 14.8 | 0.000236 | 0.014 |
| NAPSB | -0.807 | 3.34 | 14.9 | 0.000236 | 0.014 |
| PGPEP1 | -0.458 | 5.03 | 14.8 | 0.000238 | 0.014 |
| TMX4 | 0.407 | 6.85 | 14.8 | 0.000238 | 0.014 |
| FRAT2 | -0.632 | 4.71 | 14.8 | 0.000241 | 0.0141 |
| PBOV1 | 1.27 | 4.66 | 14.8 | 0.000241 | 0.0141 |
| NAA10 | -0.767 | 4.51 | 14.8 | 0.000242 | 0.0141 |
| HOTAIRM1 | 1.87 | 0.804 | 14.8 | 0.000244 | 0.0142 |
| PANK4 | -0.334 | 4.74 | 14.8 | 0.000247 | 0.0143 |
| RELL2 | -0.824 | 2.47 | 14.7 | 0.00025 | 0.0144 |
| ZNF165 | 0.918 | 3.03 | 14.7 | 0.00025 | 0.0144 |

COPD, moderate cores vs controls

| Genes | logFC | logCPM | F | PValue | FDR |
| --- | --- | --- | --- | --- | --- |
| HBA2 | 4.59 | 10.9 | 106 | 3.34E-16 | 2.88E-12 |
| HBA1 | 4.59 | 10.9 | 106 | 3.34E-16 | 2.88E-12 |
| HBB | 4.17 | 9.96 | 81.2 | 9.38E-14 | 5.38E-10 |
| FAM46C | 1.26 | 5.85 | 69.8 | 1.79E-12 | 7.7E-09 |
| GAD1 | 3.24 | -0.155 | 65.7 | 5.59E-12 | 1.92E-08 |
| ADCYAP1 | 4.88 | 0.259 | 59.9 | 2.81E-11 | 8.07E-08 |
| FCMR | 1.46 | 3.53 | 58.8 | 3.95E-11 | 9.7E-08 |
| P2RY1 | -1.17 | 4.78 | 55.1 | 1.17E-10 | 2.52E-07 |
| KRT17 | 3.02 | 5.8 | 51.9 | 3.11E-10 | 5.95E-07 |
| TIAL1 | -0.353 | 5.43 | 49.2 | 7.2E-10 | 1.13E-06 |
| MUC13 | 4.44 | 2.36 | 49.1 | 7.22E-10 | 1.13E-06 |
| CD79A | 2.23 | 3.33 | 48.6 | 8.68E-10 | 1.24E-06 |
| RNU12 | 2.98 | 6.5 | 48 | 1.05E-09 | 1.38E-06 |
| BBIP1 | 0.663 | 4.36 | 46 | 1.96E-09 | 2.41E-06 |
| CD24 | 2.19 | 5.41 | 45.7 | 2.19E-09 | 2.44E-06 |
| TRIB1 | 1.34 | 6.77 | 45.5 | 2.27E-09 | 2.44E-06 |
| MUC5AC | 4.72 | 0.911 | 45.2 | 2.56E-09 | 2.59E-06 |
| COL10A1 | 2.95 | 0.814 | 44.8 | 2.87E-09 | 2.68E-06 |
| UPK1B | 5.8 | 3.7 | 44.6 | 3.07E-09 | 2.68E-06 |
| CYR61 | 1.58 | 9.27 | 44.6 | 3.11E-09 | 2.68E-06 |
| SARAF | 0.437 | 8.4 | 43.3 | 4.72E-09 | 3.86E-06 |
| ITGBL1 | 1.27 | 5.22 | 42.4 | 6.35E-09 | 4.96E-06 |
| PTGS2 | 1.66 | 7.16 | 42.3 | 6.67E-09 | 4.99E-06 |
| B3GNT6 | 3.88 | 0.781 | 40.9 | 1.05E-08 | 6.81E-06 |
| LGALS9C | 2.77 | 1.78 | 40.9 | 1.05E-08 | 6.81E-06 |
| FHL2 | 1.33 | 3.85 | 40.9 | 1.06E-08 | 6.81E-06 |
| TXLNB | 1.55 | 2.45 | 40.8 | 1.07E-08 | 6.81E-06 |
| TUBA1A | 0.95 | 8.66 | 40.7 | 1.11E-08 | 6.81E-06 |
| HBG2 | 4.68 | 5.02 | 40.3 | 1.28E-08 | 7.54E-06 |
| HBG1 | 4.67 | 5.02 | 40.2 | 1.31E-08 | 7.54E-06 |
| MGAT4B | -0.412 | 6.49 | 39.9 | 1.5E-08 | 8.3E-06 |
| PSCA | 3.87 | 3.35 | 39.6 | 1.65E-08 | 8.85E-06 |
| INPP4B | 0.691 | 3.34 | 39 | 2.03E-08 | 1.06E-05 |
| CNTF | 2.74 | 1.11 | 38.4 | 2.45E-08 | 1.21E-05 |
| RIMKLA | -1.03 | 2.99 | 38.4 | 2.47E-08 | 1.21E-05 |
| FGGY | 0.796 | 6.63 | 38.1 | 2.71E-08 | 0.000013 |
| SERPINB5 | 3.75 | 1.93 | 37.9 | 2.88E-08 | 1.34E-05 |
| NAALADL2 | 0.891 | 3.27 | 37.6 | 3.29E-08 | 1.49E-05 |
| CD69 | 1.56 | 6.3 | 37.4 | 3.53E-08 | 1.56E-05 |
| SERPIND1 | 2.73 | 2.9 | 36.5 | 4.69E-08 | 1.97E-05 |
| TFF3 | 3.18 | 4.96 | 36.5 | 4.74E-08 | 1.97E-05 |
| DEFA3 | -4.18 | 4.21 | 36.5 | 4.82E-08 | 1.97E-05 |
| TMPRSS4 | 3.16 | 2.94 | 36.3 | 5.06E-08 | 1.99E-05 |
| DHRS9 | 2.32 | 5.17 | 36.3 | 5.19E-08 | 1.99E-05 |
| NUGGC | 1.53 | 1.71 | 36.1 | 5.4E-08 | 1.99E-05 |
| GABRP | 3.75 | 1.29 | 36.1 | 5.43E-08 | 1.99E-05 |
| PAMR1 | 2.05 | 2.49 | 36.1 | 5.45E-08 | 1.99E-05 |
| STAP1 | 1.21 | 2.14 | 36.1 | 5.54E-08 | 1.99E-05 |
| ODF3L1 | 2.13 | 0.369 | 35.8 | 6.05E-08 | 2.07E-05 |
| IL3RA | -1.07 | 4.54 | 35.8 | 6.11E-08 | 2.07E-05 |
| LGALS9B | 3 | 1.41 | 35.8 | 6.13E-08 | 2.07E-05 |
| AADAC | 1.45 | 3.42 | 35.5 | 6.74E-08 | 2.23E-05 |
| IGLL5 | 2.6 | 8.52 | 35.5 | 6.87E-08 | 2.23E-05 |
| DNALI1 | 1.48 | 5.04 | 34.9 | 8.53E-08 | 2.72E-05 |
| MYO1E | 0.665 | 6.01 | 34.6 | 9.33E-08 | 2.92E-05 |
| FAM65C | -1.64 | 2.41 | 34.5 | 9.81E-08 | 2.98E-05 |
| LOC729737 | -0.909 | 5.94 | 34.4 | 1.01E-07 | 2.98E-05 |
| SERPINB13 | 3.98 | 0.873 | 34.3 | 1.02E-07 | 2.98E-05 |
| MLF1 | 1.3 | 5.43 | 34.4 | 1.02E-07 | 2.98E-05 |
| CHL1 | 2.27 | 2.23 | 33.9 | 1.22E-07 | 3.49E-05 |
| SYTL2 | 1.05 | 4.89 | 33.7 | 1.27E-07 | 3.53E-05 |
| PCP4L1 | 1 | 3.45 | 33.7 | 1.29E-07 | 3.53E-05 |
| AMZ2P1 | 0.904 | 2.57 | 33.7 | 1.29E-07 | 3.53E-05 |
| ALAS2 | 2.86 | 0.83 | 33.6 | 1.36E-07 | 3.64E-05 |
| PHLDB3 | 1.06 | 1.63 | 33.4 | 1.42E-07 | 3.76E-05 |
| KRT23 | 2.55 | 2.51 | 32.9 | 1.74E-07 | 4.55E-05 |
| FAM102A | 0.646 | 4.66 | 32.7 | 1.82E-07 | 4.67E-05 |
| FUT8 | 0.589 | 5.82 | 32.3 | 2.11E-07 | 5.35E-05 |
| PIM2 | 1 | 5.29 | 32.3 | 2.15E-07 | 5.37E-05 |
| TARSL2 | 0.464 | 5 | 32.2 | 2.24E-07 | 5.51E-05 |
| LOC440434 | -0.426 | 7.09 | 32 | 2.35E-07 | 0.000057 |
| EGR3 | 2.03 | 4.57 | 32 | 2.39E-07 | 5.71E-05 |
| TSPAN1 | 2.62 | 6.56 | 31.9 | 2.5E-07 | 5.88E-05 |
| SPDEF | 1.95 | 2.52 | 31.8 | 2.53E-07 | 5.88E-05 |
| CHFR | -0.471 | 5.22 | 31.5 | 2.88E-07 | 6.61E-05 |
| C10orf67 | 1.41 | 4.59 | 31.2 | 3.16E-07 | 7.16E-05 |
| TNNT3 | 1.81 | 1.42 | 31 | 3.39E-07 | 7.58E-05 |
| NPEPPS | -0.311 | 7.94 | 30.7 | 3.83E-07 | 8.44E-05 |
| EGF | -1.1 | 2.09 | 30.4 | 4.27E-07 | 9.18E-05 |
| CDON | 0.82 | 3.29 | 30.4 | 4.31E-07 | 9.18E-05 |
| UGT2A1 | 3.99 | 0.955 | 30.4 | 4.32E-07 | 9.18E-05 |
| UGT2A2 | 4.01 | 0.871 | 30.3 | 4.48E-07 | 9.39E-05 |
| DAPL1 | 2.65 | 0.385 | 30.3 | 4.53E-07 | 9.39E-05 |
| BMPR1B | 1.75 | 4.39 | 30.2 | 4.6E-07 | 9.42E-05 |
| ADD3 | 0.759 | 4.95 | 30.1 | 4.77E-07 | 9.65E-05 |
| HMGCS2 | 2.64 | 2.86 | 29.9 | 5.14E-07 | 0.000103 |
| CTC-338M12.4 | 1.31 | 1.15 | 29.9 | 5.23E-07 | 0.000103 |
| SRI | 0.507 | 6.82 | 29.9 | 5.24E-07 | 0.000103 |
| ZNF208 | 1.21 | 8.12 | 29.8 | 5.41E-07 | 0.000105 |
| ANKRD36BP2 | 2.09 | 1.7 | 29.6 | 5.7E-07 | 0.000109 |
| SERPINI2 | 2.73 | 0.643 | 29.6 | 5.8E-07 | 0.00011 |
| ALDH3A1 | 2.72 | 4.37 | 29.6 | 5.87E-07 | 0.00011 |
| IRF6 | 0.959 | 4.6 | 29.5 | 6.13E-07 | 0.000113 |
| DERL3 | 1.78 | 0.538 | 29.4 | 6.32E-07 | 0.000115 |
| EYA4 | 1.49 | 4.21 | 29.4 | 6.33E-07 | 0.000115 |
| HBD | 2.26 | 2.48 | 29.3 | 6.51E-07 | 0.000117 |
| CCL28 | 2.97 | 1.81 | 29 | 7.14E-07 | 0.000127 |
| CHRNA1 | 3.63 | 1.54 | 28.9 | 7.48E-07 | 0.000131 |
| MAD1L1 | -0.394 | 5.32 | 28.8 | 7.72E-07 | 0.000134 |
| B3GNT3 | 2.39 | 1.82 | 28.5 | 8.93E-07 | 0.000154 |
| WNT10A | 2.09 | 0.285 | 28.3 | 9.43E-07 | 0.000161 |
| PTP4A3 | -0.977 | 4.11 | 28.2 | 9.88E-07 | 0.000167 |
| DEFA4 | -3.34 | 1.52 | 28 | 1.05E-06 | 0.000174 |
| ABO | 1.14 | 2.75 | 28 | 1.05E-06 | 0.000174 |
| RUNX2 | 0.905 | 1.74 | 28 | 1.06E-06 | 0.000174 |
| CD53 | 2.03 | 0.124 | 27.9 | 1.09E-06 | 0.000176 |
| WEE1 | 1.04 | 5.6 | 27.9 | 1.09E-06 | 0.000176 |
| CHAD | 2.89 | 1.01 | 27.9 | 1.1E-06 | 0.000176 |
| SLMO1 | -0.796 | 2.89 | 27.8 | 1.13E-06 | 0.000178 |
| EYA2 | 1.73 | 2.71 | 27.8 | 1.14E-06 | 0.000178 |
| CCDC74A | 2.11 | 2.55 | 27.7 | 1.21E-06 | 0.000187 |
| MSANTD4 | -0.45 | 4.08 | 27.5 | 1.3E-06 | 0.000199 |
| GBP6 | 2.8 | 2.4 | 27.4 | 1.33E-06 | 0.000203 |
| HEPACAM2 | 2.53 | 0.328 | 27.4 | 1.35E-06 | 0.000204 |
| POU2AF1 | 1.93 | 3.88 | 27.2 | 1.46E-06 | 0.000219 |
| TNS4 | 2.31 | 1.24 | 27.1 | 1.52E-06 | 0.000226 |
| VWA7 | 1.42 | 2.88 | 27 | 1.56E-06 | 0.000228 |
| KLF5 | 0.744 | 6.63 | 27 | 1.58E-06 | 0.000228 |
| TAGLN3 | 2.81 | -0.0417 | 27 | 1.58E-06 | 0.000228 |
| TRIM52 | 1.17 | 1.28 | 26.9 | 1.59E-06 | 0.000228 |
| PTGES3 | 0.449 | 8.51 | 26.9 | 1.62E-06 | 0.000229 |
| BDKRB2 | 1.74 | 1.2 | 26.9 | 1.62E-06 | 0.000229 |
| TM4SF4 | -2.48 | 1.16 | 26.7 | 1.73E-06 | 0.000242 |
| MSMB | 3.71 | 4.79 | 26.7 | 1.75E-06 | 0.000242 |
| FANK1 | 2.06 | 3.12 | 26.7 | 1.78E-06 | 0.000243 |
| TRIM29 | 2.71 | 3.47 | 26.7 | 1.78E-06 | 0.000243 |
| TSPYL1 | 0.318 | 7.14 | 26.6 | 1.82E-06 | 0.000246 |
| STAB1 | -1.12 | 4.71 | 26.4 | 1.97E-06 | 0.000262 |
| MBNL1-AS1 | 0.585 | 3.81 | 26.4 | 1.97E-06 | 0.000262 |
| CYGB | 0.798 | 4.41 | 26.4 | 1.98E-06 | 0.000262 |
| GALNT6 | 1.29 | 2.63 | 26.3 | 2.02E-06 | 0.000265 |
| MUC20 | 1.85 | 5.63 | 26.3 | 2.07E-06 | 0.000269 |
| GPR183 | 1.56 | 4.99 | 26.2 | 2.09E-06 | 0.00027 |
| FAM118A | -1.55 | 4.2 | 26.2 | 2.11E-06 | 0.000271 |
| MUC4 | 2.34 | 3.79 | 26.2 | 2.14E-06 | 0.000273 |
| SMAD3 | 0.391 | 5.82 | 26.1 | 2.18E-06 | 0.000276 |
| KBTBD4 | 0.42 | 4.97 | 26.1 | 2.24E-06 | 0.000281 |
| RPH3AL | -0.711 | 3.13 | 26 | 2.27E-06 | 0.000281 |
| TP53I3 | -0.77 | 4.27 | 26 | 2.27E-06 | 0.000281 |
| CD27 | 1.33 | 1.67 | 26 | 2.31E-06 | 0.000283 |
| SYTL3 | 0.791 | 4.46 | 25.9 | 2.36E-06 | 0.000288 |
| CACNB1 | -0.787 | 4.03 | 25.9 | 2.38E-06 | 0.000289 |
| CCDC146 | 1.59 | 5.27 | 25.8 | 2.46E-06 | 0.000296 |
| WFDC21P | 1.62 | 1.71 | 25.8 | 2.52E-06 | 0.000301 |
| TTL | -0.455 | 5.31 | 25.7 | 2.54E-06 | 0.000301 |
| ABCB6 | -0.818 | 3.05 | 25.7 | 2.6E-06 | 0.000306 |
| SLC4A8 | 1.33 | 3.24 | 25.7 | 2.62E-06 | 0.000306 |
| TP53INP2 | 0.839 | 3.66 | 25.6 | 2.67E-06 | 0.000307 |
| FUZ | 0.977 | 4.3 | 25.6 | 2.68E-06 | 0.000307 |
| GJB5 | 2.75 | -0.633 | 25.6 | 2.69E-06 | 0.000307 |
| ALDH5A1 | 0.585 | 3.69 | 25.6 | 2.7E-06 | 0.000307 |
| OSCP1 | 1.27 | 3.89 | 25.5 | 2.75E-06 | 0.000311 |
| UCP2 | 0.935 | 6.73 | 25.5 | 2.83E-06 | 0.000318 |
| SERPINB2 | 2.36 | 0.913 | 25.5 | 2.85E-06 | 0.000318 |
| IQCG | 1.63 | 5 | 25.4 | 2.87E-06 | 0.000319 |
| RRAD | 1.25 | 4.31 | 25.4 | 2.89E-06 | 0.000319 |
| CDC20B | 3.24 | 1.67 | 25.3 | 0.000003 | 0.000328 |
| EFCAB10 | 1.32 | 4.97 | 25.3 | 3.02E-06 | 0.000328 |
| RFX2 | 1.51 | 3.11 | 25.3 | 3.08E-06 | 0.000333 |
| LAMC3 | -1.14 | 4.49 | 25.2 | 3.13E-06 | 0.000336 |
| FNDC1 | 1.98 | 0.488 | 25.1 | 3.29E-06 | 0.00035 |
| DZIP3 | 1.12 | 5.75 | 25.1 | 3.31E-06 | 0.00035 |
| CXXC4 | 1.26 | 0.824 | 25.1 | 3.32E-06 | 0.00035 |
| RALGPS2 | 0.993 | 2.26 | 25 | 3.34E-06 | 0.000351 |
| GSTA5 | 2.45 | -0.0832 | 25 | 3.37E-06 | 0.000351 |
| LRP11 | 0.805 | 6.24 | 25 | 3.42E-06 | 0.000354 |
| KRT5 | 2.83 | 4.61 | 24.9 | 3.5E-06 | 0.00036 |
| TENM4 | 1.52 | 1.07 | 24.8 | 3.6E-06 | 0.000369 |
| HERC2P2 | -0.567 | 6.38 | 24.8 | 3.63E-06 | 0.000369 |
| CTGF | 1.19 | 7.94 | 24.7 | 3.76E-06 | 0.00038 |
| KIAA0125 | 1.55 | 1.45 | 24.7 | 3.78E-06 | 0.00038 |
| TRMU | -0.395 | 3.44 | 24.7 | 3.79E-06 | 0.00038 |
| DSEL | -0.745 | 4.4 | 24.7 | 3.83E-06 | 0.000381 |
| SMG9 | -0.369 | 4.77 | 24.7 | 3.85E-06 | 0.000381 |
| ADH7 | 3.29 | 3.8 | 24.7 | 3.88E-06 | 0.000381 |
| MZB1 | 2.07 | 4.44 | 24.6 | 3.92E-06 | 0.000383 |
| PLD4 | 1.4 | 1.89 | 24.6 | 4.05E-06 | 0.000394 |
| UBN1 | -0.372 | 5.18 | 24.5 | 4.11E-06 | 0.000398 |
| PAX9 | 2.88 | 0.786 | 24.5 | 4.2E-06 | 0.000403 |
| CXCR5 | 2.08 | -0.0256 | 24.4 | 4.31E-06 | 0.000412 |
| UGT1A7 | 1.95 | 0.508 | 24.4 | 4.37E-06 | 0.000415 |
| OBFC1 | -0.341 | 5.43 | 24.3 | 4.48E-06 | 0.000423 |
| GRAMD3 | 0.792 | 4.2 | 24.3 | 4.51E-06 | 0.000423 |
| TECR | 0.486 | 6.35 | 24.3 | 4.52E-06 | 0.000423 |
| CCDC66 | 0.479 | 6.45 | 24.2 | 4.63E-06 | 0.000431 |
| ZNF792 | -0.832 | 3.55 | 24.2 | 4.75E-06 | 0.000434 |
| UBE3D | 0.638 | 4.16 | 24.2 | 4.75E-06 | 0.000434 |
| SERTAD2 | 0.423 | 3.71 | 24.1 | 4.76E-06 | 0.000434 |
| ABCA13 | 2.5 | 3.01 | 24.1 | 4.77E-06 | 0.000434 |
| AK9 | 1.22 | 2.9 | 24.1 | 4.81E-06 | 0.000435 |
| USP2 | 1.81 | 2.46 | 24.1 | 4.83E-06 | 0.000435 |
| FAM213B | -0.462 | 4.63 | 24.1 | 4.87E-06 | 0.000435 |
| ALOX15 | 2.84 | 5.36 | 24.1 | 4.87E-06 | 0.000435 |
| MNS1 | 1.55 | 2.56 | 24 | 4.97E-06 | 0.000441 |
| ZBTB7C | 0.877 | 4.16 | 24 | 5.01E-06 | 0.000441 |
| CLDN10 | 2.16 | 1.87 | 24 | 5.07E-06 | 0.000441 |
| KPNA7 | 1.86 | 2.24 | 24 | 5.07E-06 | 0.000441 |
| NME5 | 2.06 | 4.26 | 24 | 5.08E-06 | 0.000441 |
| SRF | 0.502 | 5.65 | 23.9 | 5.17E-06 | 0.000446 |
| FAM174A | 0.74 | 4.2 | 23.9 | 5.21E-06 | 0.000446 |
| KIAA1211 | 1.23 | 1.83 | 23.9 | 5.22E-06 | 0.000446 |
| PLEKHG7 | 2.51 | 3.43 | 23.9 | 5.27E-06 | 0.000449 |
| ARG1 | -2.49 | 1.19 | 23.9 | 5.31E-06 | 0.00045 |
| TEX9 | 1.33 | 4.87 | 23.8 | 5.39E-06 | 0.000453 |
| REM1 | 1.01 | 2.78 | 23.8 | 5.4E-06 | 0.000453 |
| PODNL1 | 0.941 | 1.5 | 23.8 | 5.44E-06 | 0.000454 |
| KRT40 | 2.66 | 0.119 | 23.8 | 5.5E-06 | 0.000455 |
| NPTX2 | 2.44 | -0.236 | 23.8 | 5.5E-06 | 0.000455 |
| IRF2BP2 | 0.484 | 7.8 | 23.8 | 5.53E-06 | 0.000455 |
| AMN1 | 0.474 | 4.14 | 23.7 | 5.65E-06 | 0.000461 |
| CHGA | 2.95 | 0.476 | 23.7 | 5.7E-06 | 0.000461 |
| IQCK | 1.05 | 3.52 | 23.7 | 5.74E-06 | 0.000461 |
| TMEM156 | 1.08 | 1.3 | 23.7 | 5.76E-06 | 0.000461 |
| CTSV | 1.55 | 1.86 | 23.7 | 5.76E-06 | 0.000461 |
| SOX15 | 1.34 | 0.872 | 23.7 | 5.77E-06 | 0.000461 |
| JCHAIN | 1.82 | 8.23 | 23.6 | 5.95E-06 | 0.000474 |
| KLF10 | 0.959 | 6.61 | 23.6 | 6.03E-06 | 0.000478 |
| BCAS1 | 1.94 | 3.97 | 23.5 | 6.14E-06 | 0.000485 |
| GAS2 | 1.27 | 1.6 | 23.4 | 6.3E-06 | 0.000495 |
| COL17A1 | 2.43 | 1.43 | 23.3 | 6.67E-06 | 0.000522 |
| GSTA1 | 2.64 | 5.23 | 23.3 | 6.72E-06 | 0.000522 |
| SLC5A4 | -1.17 | 2.7 | 23.3 | 6.74E-06 | 0.000522 |
| SLC44A4 | 1.57 | 5.6 | 23.2 | 6.9E-06 | 0.00053 |
| S100P | 1.74 | 5.6 | 23.2 | 6.91E-06 | 0.00053 |
| BCL7A | 0.718 | 2.13 | 23.2 | 6.98E-06 | 0.000533 |
| SMOC2 | 1.11 | 4.54 | 23.1 | 7.25E-06 | 0.000552 |
| NDUFAF7 | -0.483 | 4.36 | 23 | 7.41E-06 | 0.000562 |
| CDHR4 | 2.41 | 3.23 | 23 | 7.46E-06 | 0.000563 |
| LGR5 | -1.23 | 0.927 | 23 | 7.61E-06 | 0.000572 |
| CAPS2 | 1.36 | 4 | 23 | 7.65E-06 | 0.000572 |
| LINC01207 | 2.6 | 1.54 | 22.9 | 7.77E-06 | 0.000578 |
| FUT6 | 2.67 | 0.205 | 22.9 | 7.79E-06 | 0.000578 |
| BIK | 1.25 | 0.543 | 22.9 | 7.82E-06 | 0.000578 |
| NAIP | -0.862 | 3.95 | 22.9 | 7.86E-06 | 0.000578 |
| HERC2 | -0.384 | 6.59 | 22.9 | 7.99E-06 | 0.000585 |
| ZNF132 | 0.49 | 3.36 | 22.8 | 8.16E-06 | 0.000594 |
| NOD2 | -0.865 | 1.91 | 22.8 | 8.18E-06 | 0.000594 |
| DDR1 | 0.601 | 6.52 | 22.8 | 8.25E-06 | 0.000596 |
| C12orf74 | 2.39 | 2.66 | 22.7 | 8.44E-06 | 0.000607 |
| SLC12A9 | -0.407 | 4.92 | 22.7 | 8.54E-06 | 0.000612 |
| PDLIM4 | 1.56 | 2.19 | 22.7 | 8.62E-06 | 0.000616 |
| CRIP1 | 0.909 | 5.71 | 22.6 | 8.77E-06 | 0.000624 |
| ALDH1A1 | 0.85 | 7.78 | 22.5 | 9.13E-06 | 0.000646 |
| KRT12 | 2.58 | -0.732 | 22.5 | 9.32E-06 | 0.000657 |
| RARRES2 | 0.651 | 7.41 | 22.4 | 9.41E-06 | 0.000659 |
| RNF213 | -0.448 | 6.93 | 22.4 | 9.42E-06 | 0.000659 |
| CLCN3 | 0.615 | 3.77 | 22.4 | 9.5E-06 | 0.000661 |
| CFD | 0.741 | 6.79 | 22.3 | 9.93E-06 | 0.000689 |
| C15orf57 | 0.354 | 5.24 | 22.3 | 1.01E-05 | 0.000693 |
| KLHL41 | 1.01 | 2.19 | 22.3 | 1.01E-05 | 0.000693 |
| CLDN3 | 1.09 | 4.13 | 22.3 | 1.01E-05 | 0.000693 |
| C9orf84 | 2.71 | 0.807 | 22.2 | 1.03E-05 | 0.000704 |
| P2RY10 | 1.23 | 3.17 | 22.2 | 1.03E-05 | 0.000703 |
| GSDMB | 1.98 | 1.03 | 22.2 | 1.05E-05 | 0.00071 |
| ZSCAN20 | -0.666 | 1.81 | 22.2 | 1.06E-05 | 0.000712 |
| NOB1 | -0.495 | 4.7 | 22.1 | 1.07E-05 | 0.000715 |
| CHMP1B | 0.301 | 7.1 | 22.1 | 1.07E-05 | 0.000715 |
| LINC01004 | 1.32 | 3.87 | 22.1 | 1.07E-05 | 0.000715 |
| CCL22 | 2 | 2.59 | 22.1 | 1.09E-05 | 0.000722 |
| TMEM98 | 0.39 | 5.75 | 22.1 | 0.000011 | 0.000727 |
| EGFLAM | 1.73 | -0.0992 | 22.1 | 0.000011 | 0.000728 |
| TNFRSF13B | 1.73 | 0.363 | 22 | 1.12E-05 | 0.000734 |
| SLFN13 | 1.12 | 3.5 | 22 | 1.12E-05 | 0.000734 |
| BPIFB1 | 3.28 | 9.1 | 22 | 1.14E-05 | 0.000746 |
| YPEL5 | 0.419 | 8.02 | 21.9 | 1.16E-05 | 0.000753 |
| KANSL2 | -0.385 | 4.6 | 21.8 | 1.21E-05 | 0.000784 |
| CNTD1 | 2.06 | 1.38 | 21.8 | 1.22E-05 | 0.000784 |
| MAPK8IP1 | 0.939 | 3.91 | 21.8 | 1.23E-05 | 0.000784 |
| GCFC2 | 0.426 | 3.39 | 21.8 | 1.23E-05 | 0.000784 |
| ATP13A3 | 0.425 | 6.68 | 21.8 | 1.23E-05 | 0.000784 |
| OR2A42 | -0.937 | 3.9 | 21.8 | 1.24E-05 | 0.000784 |
| OR2A1 | -0.937 | 3.9 | 21.8 | 1.24E-05 | 0.000784 |
| ZFP36L1 | 0.516 | 7.95 | 21.7 | 1.26E-05 | 0.000792 |
| CCDC173 | 1.99 | 1.72 | 21.7 | 1.26E-05 | 0.000792 |
| MAPK12 | -0.761 | 4.03 | 21.7 | 1.28E-05 | 0.000799 |
| FRZB | 1.16 | 4.19 | 21.7 | 1.29E-05 | 0.000801 |
| LOC100131496 | -0.902 | 3.21 | 21.7 | 1.29E-05 | 0.000801 |
| TSPAN8 | 1.19 | 5.38 | 21.6 | 1.31E-05 | 0.000813 |
| AACS | -0.422 | 4.3 | 21.6 | 1.33E-05 | 0.000818 |
| HTR2B | 1.55 | 0.902 | 21.6 | 1.33E-05 | 0.000818 |
| ERN2 | 2.62 | 1.64 | 21.6 | 1.35E-05 | 0.000824 |
| CCDC17 | 1.87 | 3.71 | 21.5 | 1.36E-05 | 0.000827 |
| TCEAL4 | -0.528 | 7.6 | 21.5 | 1.36E-05 | 0.000827 |
| MAPK6 | 0.494 | 5.9 | 21.5 | 1.37E-05 | 0.000827 |
| SAXO2 | 2.4 | 3.51 | 21.5 | 1.37E-05 | 0.000827 |
| S100A2 | 2.24 | 3.79 | 21.5 | 1.39E-05 | 0.000832 |
| EPHX1 | 0.606 | 7.98 | 21.5 | 0.000014 | 0.000832 |
| CEACAM5 | 2.01 | 5.26 | 21.5 | 0.000014 | 0.000832 |
| CXCL6 | 2.65 | 3.73 | 21.5 | 0.000014 | 0.000832 |
| GPAT2 | -0.903 | 3.3 | 21.4 | 1.42E-05 | 0.000843 |
| WBSCR27 | 1.68 | 1.02 | 21.4 | 1.44E-05 | 0.00085 |
| SLC41A1 | 0.394 | 4.86 | 21.4 | 1.45E-05 | 0.000855 |
| GOLM1 | 1.1 | 5.23 | 21.4 | 1.46E-05 | 0.000855 |
| FAM212A | -1.28 | 0.422 | 21.3 | 1.47E-05 | 0.000861 |
| SELE | 2.35 | 4.15 | 21.3 | 1.48E-05 | 0.000864 |
| TTLL10 | 2.2 | 1.72 | 21.3 | 1.49E-05 | 0.000866 |
| C10orf32 | 0.437 | 5.65 | 21.3 | 0.000015 | 0.000866 |
| ATP9A | 0.637 | 4.18 | 21.3 | 0.000015 | 0.000866 |
| SLC16A9 | 1.25 | 2.25 | 21.3 | 1.52E-05 | 0.000877 |

COPD, severe cores vs controls

| Genes | logFC | logCPM | F | PValue | FDR |
| --- | --- | --- | --- | --- | --- |
| HBA2 | 4.52 | 10.9 | 95.2 | 3.34E-15 | 2.88E-11 |
| HBA1 | 4.52 | 10.9 | 95.2 | 3.35E-15 | 2.88E-11 |
| TARSL2 | 0.769 | 5 | 78.7 | 1.76E-13 | 8.57E-10 |
| ITLN1 | 6.38 | 4.63 | 78.2 | 1.99E-13 | 8.57E-10 |
| TRIB1 | 1.85 | 6.77 | 77.2 | 2.6E-13 | 8.94E-10 |
| ADCYAP1 | 5.67 | 0.259 | 75.1 | 4.46E-13 | 1.28E-09 |
| HBB | 4.08 | 9.96 | 71.4 | 1.17E-12 | 2.87E-09 |
| PTGS2 | 2.03 | 7.16 | 58.3 | 4.52E-11 | 9.73E-08 |
| CYR61 | 1.86 | 9.27 | 55.2 | 1.13E-10 | 2.16E-07 |
| FAM213B | -0.765 | 4.63 | 53.7 | 1.75E-10 | 3.01E-07 |
| SLC12A9 | -0.657 | 4.92 | 50.7 | 4.48E-10 | 7.01E-07 |
| MGAT4B | -0.497 | 6.49 | 49.7 | 6.03E-10 | 8.64E-07 |
| FCMR | 1.41 | 3.53 | 49.4 | 6.74E-10 | 8.91E-07 |
| FAM46C | 1.11 | 5.85 | 48.5 | 8.79E-10 | 1.04E-06 |
| PTPN18 | -0.585 | 5.17 | 48.4 | 9.03E-10 | 1.04E-06 |
| CD69 | 1.82 | 6.3 | 48.1 | 1E-09 | 1.08E-06 |
| BBIP1 | 0.729 | 4.36 | 47.3 | 1.29E-09 | 1.3E-06 |
| RARRES2 | 0.98 | 7.41 | 46.9 | 1.45E-09 | 1.38E-06 |
| JUND | 1.18 | 7.49 | 44.4 | 3.28E-09 | 2.97E-06 |
| FGGY | 0.89 | 6.63 | 43.3 | 4.78E-09 | 4.11E-06 |
| ATL2 | 0.488 | 5.74 | 42.9 | 5.49E-09 | 4.26E-06 |
| ODF3L1 | 2.53 | 0.369 | 42.8 | 5.52E-09 | 4.26E-06 |
| ZNF208 | 1.47 | 8.12 | 42.7 | 5.7E-09 | 4.26E-06 |
| CALB2 | 2.78 | 1.77 | 41.7 | 8.06E-09 | 5.78E-06 |
| KLF10 | 1.32 | 6.61 | 40.1 | 1.37E-08 | 9.45E-06 |
| ZNF451 | 0.535 | 4.64 | 39.2 | 1.86E-08 | 1.23E-05 |
| DNASE1L3 | 2.4 | 3.72 | 39.1 | 1.94E-08 | 1.24E-05 |
| TELO2 | -0.624 | 4.35 | 38.6 | 2.33E-08 | 1.43E-05 |
| BTG2 | 1.55 | 8.08 | 38.4 | 2.45E-08 | 1.45E-05 |
| IGLL5 | 2.75 | 8.52 | 38.3 | 2.53E-08 | 1.45E-05 |
| YPEL5 | 0.58 | 8.02 | 37.8 | 3.02E-08 | 1.68E-05 |
| ABCB6 | -1.1 | 3.05 | 37.3 | 3.55E-08 | 1.91E-05 |
| RARRES3 | -0.748 | 7.72 | 37.1 | 3.9E-08 | 2.03E-05 |
| MON1A | -0.871 | 2.77 | 36.9 | 4.09E-08 | 2.07E-05 |
| SMG9 | -0.49 | 4.77 | 36.4 | 4.88E-08 | 0.000024 |
| CCDC66 | 0.613 | 6.45 | 35.2 | 7.45E-08 | 3.56E-05 |
| COL10A1 | 2.71 | 0.814 | 34.9 | 8.29E-08 | 3.85E-05 |
| CD79A | 1.87 | 3.33 | 34.5 | 9.68E-08 | 4.38E-05 |
| GPR183 | 1.83 | 4.99 | 34.4 | 1E-07 | 4.43E-05 |
| MAD1L1 | -0.461 | 5.32 | 34.1 | 1.13E-07 | 4.84E-05 |
| TCEA3 | 0.533 | 6.47 | 33.8 | 1.26E-07 | 5.29E-05 |
| DYRK3 | 1.22 | 4.39 | 33.6 | 1.34E-07 | 5.47E-05 |
| OTUD1 | 0.874 | 5.49 | 33.3 | 1.47E-07 | 5.89E-05 |
| HBG2 | 4.58 | 5.02 | 32.7 | 1.83E-07 | 7.12E-05 |
| GJA1 | 0.927 | 7.57 | 32.7 | 1.86E-07 | 7.12E-05 |
| HBG1 | 4.57 | 5.02 | 32.6 | 1.92E-07 | 7.18E-05 |
| JUNB | 1.58 | 9.01 | 32.5 | 1.96E-07 | 7.18E-05 |
| CHFR | -0.515 | 5.22 | 32.1 | 2.34E-07 | 0.000084 |
| FAM65C | -1.71 | 2.41 | 31.9 | 2.48E-07 | 8.59E-05 |
| SREBF1 | -0.739 | 6.01 | 31.9 | 2.52E-07 | 8.59E-05 |
| MBNL1-AS1 | 0.67 | 3.81 | 31.8 | 2.56E-07 | 8.59E-05 |
| TP53I3 | -0.929 | 4.27 | 31.8 | 2.6E-07 | 8.59E-05 |
| P2RY1 | -0.939 | 4.78 | 31.5 | 2.89E-07 | 9.37E-05 |
| EYA4 | 1.66 | 4.21 | 31.4 | 2.97E-07 | 9.37E-05 |
| SERPIND1 | 2.55 | 2.9 | 31.4 | 2.99E-07 | 9.37E-05 |
| CLCF1 | 1.07 | 2.27 | 30.9 | 3.54E-07 | 0.000108 |
| LAMC3 | -1.36 | 4.49 | 30.9 | 3.59E-07 | 0.000108 |
| TMEM255A | 2.23 | 1.58 | 30.6 | 4.02E-07 | 0.000118 |
| CTGF | 1.42 | 7.94 | 30.5 | 4.09E-07 | 0.000118 |
| SEMA3E | 1.28 | 3.04 | 30.5 | 4.15E-07 | 0.000118 |
| GADD45B | 1.33 | 7.39 | 30.5 | 4.19E-07 | 0.000118 |
| GPR15 | 1.96 | 2.51 | 30.4 | 4.25E-07 | 0.000118 |
| KCTD17 | -0.657 | 3.38 | 30.3 | 4.43E-07 | 0.000121 |
| PARD6B | 0.708 | 5.68 | 30.2 | 4.69E-07 | 0.000126 |
| TRAPPC2B | 0.541 | 3.76 | 30.1 | 4.88E-07 | 0.000129 |
| SELE | 2.76 | 4.15 | 30 | 5.01E-07 | 0.000129 |
| RUNX2 | 0.987 | 1.74 | 30 | 5.02E-07 | 0.000129 |
| RPS6KA1 | -0.433 | 6.19 | 29.7 | 5.6E-07 | 0.000141 |
| STAB1 | -1.27 | 4.71 | 29.7 | 5.65E-07 | 0.000141 |
| C1QTNF7 | 1.6 | 2.78 | 29.5 | 5.93E-07 | 0.000146 |
| PRKCQ-AS1 | 1.08 | 3.31 | 29.5 | 6.03E-07 | 0.000146 |
| RIF1 | 0.42 | 5.03 | 29.4 | 6.21E-07 | 0.000148 |
| INPP4B | 0.652 | 3.34 | 29.3 | 6.56E-07 | 0.000153 |
| EGR3 | 2.01 | 4.57 | 29.3 | 6.57E-07 | 0.000153 |
| HK3 | -1.04 | 5.25 | 29 | 7.19E-07 | 0.000165 |
| CHRNA1 | 3.84 | 1.54 | 29 | 7.27E-07 | 0.000165 |
| KEAP1 | -0.337 | 5.21 | 28.8 | 7.73E-07 | 0.000173 |
| JCHAIN | 1.99 | 8.23 | 28.8 | 7.88E-07 | 0.000174 |
| LDLR | 0.983 | 8.53 | 28.7 | 8.17E-07 | 0.000178 |
| IRAK1 | -0.743 | 3.39 | 28.6 | 8.47E-07 | 0.000182 |
| SURF6 | -0.543 | 5.51 | 28.6 | 8.6E-07 | 0.000183 |
| TMEM8A | -0.514 | 5.45 | 28.4 | 8.98E-07 | 0.000188 |
| ALAS2 | 2.77 | 0.83 | 28.4 | 9.23E-07 | 0.000191 |
| BLID | 1.26 | 2.32 | 28.3 | 9.58E-07 | 0.000196 |
| PIM2 | 0.986 | 5.29 | 28 | 1.08E-06 | 0.000218 |
| AMZ2P1 | 0.883 | 2.57 | 27.7 | 1.18E-06 | 0.000236 |
| HBD | 2.34 | 2.48 | 27.7 | 1.21E-06 | 0.000238 |
| KLRC1 | 1.26 | 1.75 | 27.6 | 1.23E-06 | 0.000238 |
| ZNF707 | -0.563 | 4.17 | 27.6 | 1.24E-06 | 0.000238 |
| DUSP2 | 1.51 | 2.75 | 27.6 | 1.25E-06 | 0.000238 |
| NNMT | 1.31 | 7.37 | 27.5 | 1.28E-06 | 0.000242 |
| FAM109B | -0.596 | 3.44 | 27.4 | 1.31E-06 | 0.000245 |
| EGR2 | 1.67 | 6.08 | 27.3 | 1.4E-06 | 0.000258 |
| WIPI1 | -0.435 | 4.68 | 27.2 | 1.42E-06 | 0.00026 |
| DCAF16 | 0.44 | 4.93 | 27.2 | 1.45E-06 | 0.000263 |
| RCC1 | -0.681 | 4.27 | 26.9 | 1.62E-06 | 0.00029 |
| VMP1 | 0.861 | 8.4 | 26.9 | 1.65E-06 | 0.000292 |
| SNPH | -0.996 | 2.35 | 26.6 | 1.8E-06 | 0.000316 |
| GCFC2 | 0.51 | 3.39 | 26.5 | 1.92E-06 | 0.000334 |
| CCDC14 | 0.839 | 4.63 | 26.3 | 2.02E-06 | 0.000342 |
| CDKAL1 | 0.445 | 6.58 | 26.3 | 2.02E-06 | 0.000342 |
| TOX4 | 0.336 | 6.15 | 26.3 | 2.03E-06 | 0.000342 |
| TSPAN14 | -0.547 | 6.58 | 26.3 | 2.06E-06 | 0.000344 |
| CRYBB2P1 | -0.802 | 2.71 | 26.1 | 2.21E-06 | 0.000364 |
| LDHB | -0.476 | 8.1 | 26.1 | 2.22E-06 | 0.000364 |
| MIR100HG | 1.11 | 2.46 | 26 | 2.29E-06 | 0.000368 |
| PTP4A3 | -1.01 | 4.11 | 26 | 2.29E-06 | 0.000368 |
| AACS | -0.507 | 4.3 | 26 | 2.33E-06 | 0.000368 |
| NPIPA7 | -0.743 | 4.82 | 25.9 | 2.37E-06 | 0.000368 |
| NPIPA8 | -0.743 | 4.82 | 25.9 | 2.37E-06 | 0.000368 |
| DAPL1 | 2.69 | 0.385 | 25.9 | 2.37E-06 | 0.000368 |
| GAS2 | 1.5 | 1.6 | 25.8 | 2.44E-06 | 0.000375 |
| PTP4A1 | 0.783 | 7.21 | 25.8 | 2.46E-06 | 0.000375 |
| C11orf96 | 1.28 | 6.75 | 25.6 | 2.65E-06 | 0.000401 |
| SNHG20 | -0.72 | 3.7 | 25.6 | 2.73E-06 | 0.000408 |
| MTG2 | -0.522 | 2.88 | 25.5 | 2.76E-06 | 0.000409 |
| IMPDH1 | -0.629 | 5.52 | 25.3 | 0.000003 | 0.000439 |
| HMGCS2 | 2.58 | 2.86 | 25.3 | 3.01E-06 | 0.000439 |
| TRIL | 1.33 | 3.08 | 25.3 | 3.04E-06 | 0.000439 |
| STAM | 0.455 | 5.16 | 25.2 | 3.16E-06 | 0.000447 |
| ZNF140 | 0.423 | 4.76 | 25.2 | 3.17E-06 | 0.000447 |
| ZXDA | 0.617 | 3.14 | 25.2 | 3.17E-06 | 0.000447 |
| PDHX | 0.457 | 5.54 | 25 | 3.36E-06 | 0.00047 |
| HLA-G | 2.76 | 5.67 | 24.9 | 3.52E-06 | 0.000484 |
| ATP13A3 | 0.486 | 6.68 | 24.9 | 3.53E-06 | 0.000484 |
| PDXP | -0.565 | 2.59 | 24.9 | 3.58E-06 | 0.000484 |
| ANKRD28 | 0.561 | 4.13 | 24.9 | 3.58E-06 | 0.000484 |
| KRT16P1 | 2.92 | 3.27 | 24.8 | 3.62E-06 | 0.000484 |
| DNAJB4 | 0.749 | 6.51 | 24.8 | 3.63E-06 | 0.000484 |
| RORB | -1.09 | 2.4 | 24.6 | 3.91E-06 | 0.000517 |
| PPP1R15B | 0.68 | 6.16 | 24.6 | 3.97E-06 | 0.000522 |
| PLA2G2D | 3.03 | 0.996 | 24.6 | 4.03E-06 | 0.000525 |
| DEFA3 | -3.77 | 4.21 | 24.5 | 4.09E-06 | 0.000529 |
| NHSL1 | 0.692 | 4.51 | 24.5 | 4.2E-06 | 0.000535 |
| LYL1 | -0.862 | 3.73 | 24.5 | 4.21E-06 | 0.000535 |
| ATXN7 | 1.06 | 2.54 | 24.4 | 4.26E-06 | 0.000535 |
| SLC4A3 | -1.4 | 1.56 | 24.4 | 4.29E-06 | 0.000535 |
| CDAN1 | -0.651 | 3.28 | 24.4 | 4.31E-06 | 0.000535 |
| ADAMTS15 | -1 | 4.31 | 24.4 | 4.32E-06 | 0.000535 |
| RHBDD3 | -0.852 | 3.87 | 24.3 | 4.43E-06 | 0.000544 |
| DBNDD1 | -0.775 | 2.47 | 24.3 | 4.46E-06 | 0.000544 |
| SMG5 | -0.393 | 6.41 | 24.2 | 4.71E-06 | 0.00057 |
| ARL4C | 0.855 | 4.25 | 24.1 | 4.77E-06 | 0.000574 |
| DUSP1 | 1.22 | 11 | 24.1 | 4.81E-06 | 0.000575 |
| RAB13 | -0.458 | 7.18 | 24 | 5.01E-06 | 0.000595 |
| SLMO1 | -0.799 | 2.89 | 24 | 5.12E-06 | 0.000603 |
| CFD | 0.792 | 6.79 | 23.9 | 5.16E-06 | 0.000604 |
| MBNL1 | 0.376 | 5.03 | 23.8 | 5.45E-06 | 0.000634 |
| REM1 | 1.11 | 2.78 | 23.7 | 5.66E-06 | 0.000654 |
| KLHDC8B | -0.57 | 4.2 | 23.6 | 5.83E-06 | 0.000665 |
| PPP1R15A | 1.13 | 5.82 | 23.6 | 5.87E-06 | 0.000665 |
| CECR5 | -0.434 | 4.24 | 23.6 | 5.91E-06 | 0.000665 |
| RELL2 | -0.822 | 2.47 | 23.6 | 5.91E-06 | 0.000665 |
| H1F0 | -0.76 | 6.56 | 23.5 | 6.07E-06 | 0.000678 |
| PAMR1 | 1.84 | 2.49 | 23.5 | 6.23E-06 | 0.000691 |
| EXOSC1 | 0.478 | 6.73 | 23.4 | 6.32E-06 | 0.000696 |
| TTL | -0.467 | 5.31 | 23.4 | 6.41E-06 | 0.000702 |
| CENPO | -0.653 | 3.23 | 23.4 | 6.46E-06 | 0.000703 |
| SPECC1 | -0.43 | 3.91 | 23.4 | 6.5E-06 | 0.000703 |
| GADD45G | -0.876 | 3.52 | 23.3 | 6.68E-06 | 0.000717 |
| SLC4A2 | -0.423 | 5.37 | 23.3 | 6.71E-06 | 0.000717 |
| SYNDIG1L | 1.37 | 1.89 | 23.3 | 6.78E-06 | 0.00072 |
| MYC | 1.34 | 5.43 | 23.2 | 6.88E-06 | 0.000726 |
| ARHGAP15 | 0.617 | 4.22 | 23.2 | 6.94E-06 | 0.000728 |
| BCLAF1 | 0.303 | 6.63 | 23.2 | 7.05E-06 | 0.000735 |
| CCSER1 | 0.795 | 3.46 | 23.1 | 7.22E-06 | 0.000748 |
| BCAT2 | -0.579 | 6.44 | 22.9 | 7.75E-06 | 0.000798 |
| CTNS | -0.468 | 4.53 | 22.9 | 7.85E-06 | 0.000803 |
| TAF7 | 0.674 | 6.83 | 22.9 | 7.98E-06 | 0.000812 |
| SLC26A6 | -0.653 | 3.36 | 22.8 | 8.3E-06 | 0.000835 |
| CTC-338M12.4 | 1.25 | 1.15 | 22.8 | 8.3E-06 | 0.000835 |
| KIF15 | -1.2 | 1.81 | 22.6 | 8.79E-06 | 0.000879 |
| TMEM98 | 0.421 | 5.75 | 22.6 | 8.99E-06 | 0.000894 |
| ZNF581 | -0.773 | 3.18 | 22.5 | 9.29E-06 | 0.000914 |
| POLR2H | -0.383 | 5.46 | 22.5 | 9.33E-06 | 0.000914 |
| RASGEF1B | 0.745 | 6.48 | 22.5 | 9.35E-06 | 0.000914 |
| ITGBL1 | 0.978 | 5.22 | 22.3 | 0.00001 | 0.000976 |
| SLC41A1 | 0.429 | 4.86 | 22.2 | 1.02E-05 | 0.000987 |
| C15orf57 | 0.378 | 5.24 | 22.2 | 1.05E-05 | 0.00101 |
| SRF | 0.514 | 5.65 | 22.2 | 1.05E-05 | 0.00101 |
| STAP1 | 1.02 | 2.14 | 22.1 | 1.07E-05 | 0.00102 |
| GK3P | 0.893 | 2.06 | 22.1 | 0.000011 | 0.00104 |
| ADCYAP1R1 | -1.92 | 0.802 | 22.1 | 0.000011 | 0.00104 |
| NAE1 | 0.375 | 5.14 | 22 | 1.12E-05 | 0.00105 |
| TRIM52 | 1.14 | 1.28 | 21.9 | 1.16E-05 | 0.00108 |
| SLC6A14 | 0.893 | 5.23 | 21.9 | 1.16E-05 | 0.00108 |
| NR4A3 | 1.87 | 5.35 | 21.9 | 1.17E-05 | 0.00108 |
| SOCS2 | 2.07 | 4.64 | 21.9 | 1.18E-05 | 0.00108 |
| AADAC | 1.22 | 3.42 | 21.9 | 1.19E-05 | 0.00108 |
| DUSP26 | 1.44 | 1.18 | 21.9 | 1.19E-05 | 0.00108 |
| SLC28A3 | 1.31 | 2.04 | 21.8 | 1.21E-05 | 0.00109 |
| DOCK4 | 0.525 | 5.65 | 21.8 | 1.22E-05 | 0.00109 |
| CD53 | 1.87 | 0.124 | 21.8 | 1.24E-05 | 0.0011 |
| NFKBIZ | 1.44 | 8.64 | 21.8 | 1.24E-05 | 0.0011 |
| KLF11 | 0.567 | 6.16 | 21.8 | 1.25E-05 | 0.0011 |
| SPIC | -2.44 | 0.327 | 21.7 | 1.26E-05 | 0.0011 |
| TMX4 | 0.398 | 6.85 | 21.7 | 1.26E-05 | 0.0011 |
| CRYL1 | 0.46 | 4.73 | 21.7 | 1.28E-05 | 0.00111 |
| RNPEPL1 | -0.355 | 7.24 | 21.7 | 1.28E-05 | 0.00111 |
| TCEAL3 | -0.538 | 5.69 | 21.6 | 1.31E-05 | 0.00112 |
| ESRRA | -0.492 | 4.23 | 21.6 | 1.34E-05 | 0.00114 |
| DUSP5 | 1.17 | 4.41 | 21.6 | 1.35E-05 | 0.00114 |
| IL2 | 1.81 | -0.105 | 21.6 | 1.35E-05 | 0.00114 |
| CXCL3 | 1.95 | 5.56 | 21.5 | 0.000014 | 0.00118 |
| SRBD1 | 0.426 | 5.88 | 21.4 | 1.41E-05 | 0.00118 |
| SNCA | 0.696 | 3.91 | 21.5 | 1.41E-05 | 0.00118 |
| LINC01004 | 1.36 | 3.87 | 21.5 | 1.41E-05 | 0.00118 |
| IDI1 | 0.503 | 6.83 | 21.4 | 1.43E-05 | 0.00118 |
| CCNT1 | 0.319 | 5.55 | 21.4 | 1.44E-05 | 0.00118 |
| NLRP12 | -1.2 | 2.04 | 21.4 | 1.44E-05 | 0.00118 |
| NTN4 | 0.742 | 7.86 | 21.4 | 1.46E-05 | 0.00119 |
| RASGRF2 | 0.849 | 2.46 | 21.4 | 1.46E-05 | 0.00119 |
| STEAP4 | 0.96 | 7.42 | 21.3 | 1.48E-05 | 0.00119 |
| IP6K2 | 0.512 | 4.74 | 21.3 | 1.49E-05 | 0.0012 |
| NPIPA1 | -0.651 | 4.59 | 21.3 | 1.52E-05 | 0.00122 |
| PEX13 | 0.318 | 5.6 | 21.2 | 1.54E-05 | 0.00122 |
| MIR5096 | 0.763 | 1.79 | 21.2 | 1.57E-05 | 0.00124 |
| ATXN7L3 | -0.357 | 5.91 | 21.1 | 0.000016 | 0.00126 |
| NPM3 | -0.718 | 3.84 | 21.1 | 1.64E-05 | 0.00129 |
| KLF17 | -1.91 | 0.419 | 21 | 1.69E-05 | 0.00132 |
| C9orf64 | -0.438 | 4.71 | 21 | 1.71E-05 | 0.00133 |
| TCEAL4 | -0.553 | 7.6 | 21 | 1.73E-05 | 0.00133 |
| SLC19A2 | 1.02 | 4.07 | 20.9 | 1.74E-05 | 0.00133 |
| GDF11 | -0.886 | 2.05 | 20.9 | 1.74E-05 | 0.00133 |
| CORO1B | -0.363 | 6.26 | 20.9 | 1.75E-05 | 0.00133 |
| IER3 | 1.33 | 5.69 | 20.9 | 1.75E-05 | 0.00133 |
| IKBKG | -1.77 | 0.855 | 20.9 | 1.77E-05 | 0.00134 |
| NT5DC2 | -0.546 | 5.27 | 20.9 | 1.79E-05 | 0.00135 |
| CCL5 | 1.18 | 5.64 | 20.8 | 1.81E-05 | 0.00136 |
| SNORA70G | 0.507 | 6.14 | 20.8 | 1.83E-05 | 0.00136 |
| SMARCD3 | -0.531 | 4.38 | 20.8 | 1.83E-05 | 0.00136 |
| AQP1 | -0.941 | 10.3 | 20.8 | 1.85E-05 | 0.00138 |
| SDR42E1 | -0.59 | 3.04 | 20.7 | 1.89E-05 | 0.00139 |
| ZNF253 | 0.581 | 5.36 | 20.7 | 1.89E-05 | 0.00139 |
| SH3BP2 | -0.748 | 3.07 | 20.7 | 0.000019 | 0.00139 |
| TSPAN17 | -0.409 | 4.25 | 20.7 | 1.95E-05 | 0.00142 |
| BAX | -0.548 | 5.85 | 20.6 | 1.99E-05 | 0.00144 |
| RPL32 | 0.372 | 9.25 | 20.6 | 0.00002 | 0.00144 |
| DDIT4 | -1.21 | 6.59 | 20.6 | 2.03E-05 | 0.00146 |
| FAM118A | -1.52 | 4.2 | 20.6 | 2.04E-05 | 0.00146 |
| HGH1 | -0.75 | 3.49 | 20.5 | 2.05E-05 | 0.00147 |
| DTYMK | -0.683 | 3.05 | 20.5 | 2.11E-05 | 0.0015 |
| CLN3 | -0.344 | 5.61 | 20.4 | 2.16E-05 | 0.00153 |
| FLT4 | -0.926 | 6 | 20.4 | 2.16E-05 | 0.00153 |
| BMP2 | 1.18 | 5.41 | 20.4 | 2.18E-05 | 0.00153 |
| ZBTB10 | 0.415 | 4.39 | 20.4 | 2.19E-05 | 0.00153 |
| DDX23 | -0.357 | 5.32 | 20.3 | 2.23E-05 | 0.00156 |
| ATP6V1B1 | 1.37 | 1.72 | 20.3 | 2.26E-05 | 0.00157 |
| YARS | -0.682 | 5.88 | 20.3 | 2.31E-05 | 0.0016 |
| NAIP | -0.875 | 3.95 | 20.2 | 2.32E-05 | 0.0016 |
| ATF3 | 1.96 | 7.05 | 20.2 | 2.36E-05 | 0.00162 |
| OBFC1 | -0.334 | 5.43 | 20.2 | 2.37E-05 | 0.00162 |
| RPL10 | 0.452 | 9.7 | 20.1 | 2.48E-05 | 0.00168 |
| AJAP1 | -1.8 | 2.48 | 20.1 | 2.51E-05 | 0.0017 |
| HTR2B | 1.57 | 0.902 | 20 | 2.54E-05 | 0.00171 |
| RPS27A | 0.365 | 9.22 | 20 | 2.56E-05 | 0.00172 |
| SLC27A3 | -0.64 | 5.72 | 20 | 2.58E-05 | 0.00172 |
| TIMM22 | -0.42 | 3.6 | 20 | 0.000026 | 0.00173 |
| ZNF597 | 0.733 | 2.43 | 20 | 2.61E-05 | 0.00173 |
| CYGB | 0.744 | 4.41 | 19.9 | 2.62E-05 | 0.00174 |
| TNFRSF13B | 1.69 | 0.363 | 19.9 | 2.67E-05 | 0.00176 |
| NUGGC | 1.22 | 1.71 | 19.9 | 2.68E-05 | 0.00176 |
| LINC00936 | 1.08 | 2.16 | 19.9 | 0.000027 | 0.00177 |
| ZRANB1 | 0.373 | 5.8 | 19.8 | 2.74E-05 | 0.00179 |
| RANBP2 | 0.272 | 7.52 | 19.8 | 2.76E-05 | 0.00179 |
| IL6 | 2.08 | 5.65 | 19.8 | 2.81E-05 | 0.00181 |
| KLF5 | 0.702 | 6.63 | 19.8 | 2.85E-05 | 0.00183 |
| ALOX12 | -0.45 | 4.22 | 19.8 | 2.85E-05 | 0.00183 |
| CDC25B | -0.717 | 6.49 | 19.7 | 2.92E-05 | 0.00187 |
| POLI | 0.501 | 4.49 | 19.7 | 2.94E-05 | 0.00187 |
| H3F3B | 0.405 | 9.82 | 19.6 | 3.04E-05 | 0.00193 |
| MED12L | -1.74 | -0.195 | 19.6 | 3.05E-05 | 0.00193 |
| C2CD4B | 1.64 | 3.61 | 19.6 | 3.06E-05 | 0.00193 |
| SESTD1 | 0.543 | 5.66 | 19.6 | 3.09E-05 | 0.00194 |
| USP35 | -0.525 | 3.41 | 19.5 | 3.11E-05 | 0.00194 |
| PARP8 | 0.38 | 5.27 | 19.5 | 3.16E-05 | 0.00197 |
| ARL5B | 0.656 | 5.42 | 19.5 | 0.000032 | 0.00199 |
| QTRTD1 | -0.41 | 4.11 | 19.5 | 3.23E-05 | 0.002 |
| TSC22D2 | 1.34 | 0.836 | 19.4 | 3.27E-05 | 0.00202 |
| FREM1 | 0.817 | 2.71 | 19.4 | 3.33E-05 | 0.00204 |
| ERRFI1 | 1.33 | 2.22 | 19.4 | 3.35E-05 | 0.00205 |
| ABCB1 | 1.15 | 2.06 | 19.3 | 3.46E-05 | 0.0021 |
| LINC00312 | 1.17 | 4.44 | 19.3 | 3.47E-05 | 0.0021 |
| DDX3X | 0.484 | 8.17 | 19.3 | 3.47E-05 | 0.0021 |
| EGF | -0.952 | 2.09 | 19.3 | 3.48E-05 | 0.0021 |
| EGR1 | 1.79 | 8.57 | 19.2 | 3.53E-05 | 0.00212 |
| C19orf25 | -0.385 | 3.46 | 19.2 | 3.56E-05 | 0.00214 |
| PPP1R10 | 0.641 | 7.28 | 19.2 | 3.59E-05 | 0.00214 |
| LYPLAL1 | 0.578 | 5.54 | 19.2 | 0.000036 | 0.00214 |
| EIF1 | 0.456 | 9.86 | 19.2 | 0.000036 | 0.00214 |
| ADAM15 | -0.54 | 7.51 | 19.1 | 3.78E-05 | 0.00223 |
| CD27 | 1.19 | 1.67 | 19 | 3.83E-05 | 0.00223 |
| VASH1 | -0.666 | 4.86 | 19 | 3.83E-05 | 0.00223 |
| FOXA2 | 0.697 | 2.01 | 19 | 3.83E-05 | 0.00223 |
| NAALADL2 | 0.707 | 3.27 | 19 | 3.84E-05 | 0.00223 |
| ADAMTS2 | -0.893 | 3.52 | 19 | 3.84E-05 | 0.00223 |
| JMJD1C | 0.642 | 6.74 | 19 | 3.86E-05 | 0.00224 |
| PPM1F | -0.67 | 5.11 | 19 | 3.88E-05 | 0.00224 |
| CCDC115 | 0.355 | 4.6 | 19 | 0.000039 | 0.00224 |

**Table E2** : Enrichment scores for xCell cell types comparison

| **Cells** |  |  |  |  |  |  |
| --- | --- | --- | --- | --- | --- | --- |
|  | ***COPD, mild cores vs CT*** | | ***COPD, moderate cores vs CT*** | | ***COPD, severe cores vs CT*** | |
|  | LogFC | adj p-value | LogFC | adj p-value | LogFC | adj p-value |
| CD8+ T cells | 5.06 | **0.031** | 1.19 | 0.731 | 2.06 | 0.571 |
| Naive CD8+ T cells | 1.06 | 0.788 | 1.27 | 0.385 | 1.28 | 0.352 |
| naive B cells | 1.56 | 0.361 | 0.34 | 0.859 | 0.04 | 0.991 |
| Dentritic cells | -0.27 | 0.939 | 2.13 | 0.561 | 2.19 | 0.517 |
| Monocytes | -1.90 | 0.838 | -2.47 | 0.327 | 0.44 | 0.884 |
| Macrophages | -1.32 | 0.908 | -0.58 | 0.874 | -0.81 | 0.870 |
| B cells | 1.82 | 0.858 | 4.17 | 0.113 | 3.91 | 0.156 |
| Naive CD4+ cells | 0.38 | 0.939 | 0.161 | 0.978 | 1.09 | 0.747 |
| CD4+ memory T cells | 2.45 | 0.361 | 1.23 | 0.660 | 4.30 | **0.004** |
| Th1 cells | -0.10 | 0.95 | -0.08 | 0.978 | 0.37 | 0.884 |
| Th2 cells | 1.85 | 0.838 | 2.00 | 0.605 | 1.63 | 0.655 |
| Tregs cells | 0.280 | 0.908 | 0.06 | 0.978 | 1.53 | **0.095** |
| Plasma cells | -1.15 | 0.908 | 0.58 | 0.874 | 1.59 | 0.747 |
| Class switch B cells | 0.87 | 0.897 | 2.21 | 0.202 | 1.93 | 0.343 |
| Fibroblasts | -0.63 | 0.908 | 0.28 | 0.974 | 0.96 | 0.858 |
| Neutrophils | 1.01 | 0.908 | -0.93 | 0.783 |  | 0.884 |
| Eosinophils | -0.11 | 0.939 | -0.23 | 0.874 |  | 0.870 |
| Basophils | 1.79 | 0.858 | -0.93 | 0.763 |  | 0.884 |

**Table E3**: KEGG-GSEA pathways enrichment scores (ES) comparison

***COPD, mild cores vs CT***

| geneSet | description | ES | FDR |
| --- | --- | --- | --- |
| hsa04740 | Olfactory transduction | 0.802607 | **0** |
| hsa05144 | Malaria | 0.667404 | **0.005689** |
| hsa05143 | African trypanosomiasis | 0.697452 | **0.011108** |
| hsa00860 | Porphyrin and chlorophyll metabolism | 0.665313 | **0.011216** |
| hsa00053 | Ascorbate and aldarate metabolism | 0.744802 | **0.012394** |
| hsa05204 | Chemical carcinogenesis | 0.607566 | **0.012733** |
| hsa00980 | Metabolism of xenobiotics by cytochrome P450 | 0.604097 | **0.0173** |
| hsa04672 | Intestinal immune network for IgA production | 0.630045 | **0.019867** |
| hsa00040 | Pentose and glucuronate interconversions | 0.659781 | **0.021843** |
| hsa05323 | Rheumatoid arthritis | 0.5697 | **0.021863** |
| hsa00982 | Drug metabolism | 0.580686 | **0.031845** |
| hsa05320 | Autoimmune thyroid disease | 0.605331 | **0.036448** |
| hsa04940 | Type I diabetes mellitus | 0.621922 | **0.036506** |
| hsa05332 | Graft-versus-host disease | 0.610557 | **0.044178** |
| hsa00830 | Retinol metabolism | 0.579288 | **0.048114** |
| hsa04060 | Cytokine-cytokine receptor interaction | 0.489215 | **0.064308** |
| hsa04657 | IL-17 signaling pathway | 0.522021 | **0.084334** |
| hsa03440 | Homologous recombination | -0.54318 | **0.084438** |
| hsa00140 | Steroid hormone biosynthesis | 0.555226 | 0.10972 |
| hsa00532 | Glycosaminoglycan biosynthesis | -0.64183 | 0.114875 |
| hsa00670 | One carbon pool by folate | -0.60066 | 0.124104 |
| hsa04668 | TNF signaling pathway | 0.489613 | 0.124307 |
| hsa00601 | Glycosphingolipid biosynthesis | 0.620266 | 0.126401 |
| hsa04630 | JAK-STAT signaling pathway | 0.482408 | 0.127032 |
| hsa03008 | Ribosome biogenesis in eukaryotes | -0.47393 | 0.127639 |
| hsa03010 | Ribosome | -0.38496 | 0.134266 |
| hsa00970 | Aminoacyl-tRNA biosynthesis | -0.52832 | 0.153166 |
| hsa04611 | Platelet activation | -0.41727 | 0.154639 |
| hsa03050 | Proteasome | -0.46242 | 0.158839 |
| hsa01040 | Biosynthesis of unsaturated fatty acids | -0.50114 | 0.161839 |
| hsa00052 | Galactose metabolism | -0.51979 | 0.164294 |
| hsa03030 | DNA replication | -0.47526 | 0.167501 |
| hsa00790 | Folate biosynthesis | -0.52644 | 0.167818 |
| hsa04978 | Mineral absorption | -0.42974 | 0.175838 |
| hsa05330 | Allograft rejection | 0.558846 | 0.187497 |
| hsa05031 | Amphetamine addiction | 0.522206 | 0.190515 |
| hsa04110 | Cell cycle | -0.35919 | 0.206887 |
| hsa00592 | alpha-Linolenic acid metabolism | -0.50976 | 0.208804 |
| hsa04915 | Estrogen signaling pathway | 0.47171 | 0.212837 |
| hsa00220 | Arginine biosynthesis | -0.5039 | 0.230789 |
| hsa05030 | Cocaine addiction | 0.545246 | 0.232726 |
| hsa04966 | Collecting duct acid secretion | 0.593498 | 0.234526 |
| hsa05132 | Salmonella infection | 0.479503 | 0.240369 |
| hsa04666 | Fc gamma R-mediated phagocytosis | -0.36877 | 0.242391 |
| hsa04350 | TGF-beta signaling pathway | 0.476079 | 0.244356 |
| hsa00983 | Drug metabolism | 0.484578 | 0.248549 |
| hsa05142 | Chagas disease (American trypanosomiasis) | 0.465206 | 0.256096 |
| hsa00515 | Mannose type O-glycan biosynthesis | -0.49186 | 0.281927 |
| hsa01210 | 2-Oxocarboxylic acid metabolism | -0.49377 | 0.283802 |
| hsa04623 | Cytosolic DNA-sensing pathway | 0.480825 | 0.28821 |
| hsa04512 | ECM-receptor interaction | -0.34872 | 0.295925 |
| hsa03450 | Non-homologous end-joining | -0.57052 | 0.296951 |
| hsa00450 | Selenocompound metabolism | -0.50922 | 0.307016 |
| hsa03460 | Fanconi anemia pathway | -0.37329 | 0.309903 |
| hsa00510 | N-Glycan biosynthesis | -0.37593 | 0.367125 |
| hsa01212 | Fatty acid metabolism | -0.36194 | 0.390614 |
| hsa04620 | Toll-like receptor signaling pathway | 0.443774 | 0.390905 |
| hsa00630 | Glyoxylate and dicarboxylate metabolism | -0.43179 | 0.397115 |
| hsa04914 | Progesterone-mediated oocyte maturation | -0.31649 | 0.408153 |
| hsa00564 | Glycerophospholipid metabolism | -0.3152 | 0.410204 |
| hsa04664 | Fc epsilon RI signaling pathway | -0.33542 | 0.423943 |
| hsa05416 | Viral myocarditis | 0.46664 | 0.452354 |
| hsa04270 | Vascular smooth muscle contraction | -0.30469 | 0.453543 |
| hsa00604 | Glycosphingolipid biosynthesis | -0.4698 | 0.483242 |
| hsa00561 | Glycerolipid metabolism | -0.33778 | 0.488027 |
| hsa05100 | Bacterial invasion of epithelial cells | -0.31958 | 0.493979 |
| hsa00240 | Pyrimidine metabolism | -0.29721 | 0.494901 |
| hsa04974 | Protein digestion and absorption | -0.31698 | 0.495766 |
| hsa04510 | Focal adhesion | -0.26882 | 0.501877 |
| hsa00380 | Tryptophan metabolism | -0.3565 | 0.508051 |
| hsa01230 | Biosynthesis of amino acids | -0.31992 | 0.508909 |
| hsa04370 | VEGF signaling pathway | -0.30374 | 0.518661 |
| hsa00051 | Fructose and mannose metabolism | -0.36835 | 0.521303 |
| hsa04610 | Complement and coagulation cascades | 0.440124 | 0.580362 |
| hsa04514 | Cell adhesion molecules (CAMs) | 0.414411 | 0.582786 |
| hsa05160 | Hepatitis C | 0.406625 | 0.58924 |
| hsa05133 | Pertussis | 0.438148 | 0.591317 |
| hsa05164 | Influenza A | 0.401144 | 0.591458 |
| hsa04928 | Parathyroid hormone synthesis. secretion and action | 0.424066 | 0.59256 |
| hsa00520 | Amino sugar and nucleotide sugar metabolism | -0.31789 | 0.595328 |
| hsa05212 | Pancreatic cancer | -0.29314 | 0.598967 |
| hsa00480 | Glutathione metabolism | -0.30643 | 0.61157 |
| hsa05321 | Inflammatory bowel disease (IBD) | 0.43919 | 0.616278 |
| hsa04062 | Chemokine signaling pathway | 0.390563 | 0.629692 |
| hsa05167 | Kaposi sarcoma-associated herpesvirus infection | 0.39322 | 0.631482 |
| hsa04530 | Tight junction | 0.400492 | 0.640578 |
| hsa00340 | Histidine metabolism | 0.534429 | 0.64158 |
| hsa04640 | Hematopoietic cell lineage | 0.413739 | 0.641785 |
| hsa04730 | Long-term depression | -0.30157 | 0.659794 |
| hsa04216 | Ferroptosis | 0.46097 | 0.668359 |
| hsa04923 | Regulation of lipolysis in adipocytes | 0.441064 | 0.67264 |
| hsa04918 | Thyroid hormone synthesis | -0.29121 | 0.674674 |
| hsa05161 | Hepatitis B | 0.38611 | 0.679742 |
| hsa05120 | Epithelial cell signaling in Helicobacter pylori infection | 0.416814 | 0.681367 |
| hsa04924 | Renin secretion | 0.430226 | 0.681801 |
| hsa05168 | Herpes simplex infection | 0.379054 | 0.683966 |
| hsa04080 | Neuroactive ligand-receptor interaction | 0.383454 | 0.685419 |
| hsa05163 | Human cytomegalovirus infection | 0.375021 | 0.687939 |
| hsa05310 | Asthma | 0.484352 | 0.689968 |
| hsa00600 | Sphingolipid metabolism | 0.445637 | 0.69145 |
| hsa00310 | Lysine degradation | -0.29482 | 0.695524 |
| hsa05150 | Staphylococcus aureus infection | 0.430336 | 0.696552 |
| hsa00590 | Arachidonic acid metabolism | -0.29013 | 0.704081 |
| hsa05418 | Fluid shear stress and atherosclerosis | 0.376256 | 0.710595 |
| hsa00130 | Ubiquinone and other terpenoid-quinone biosynthesis | -0.43856 | 0.728318 |
| hsa04975 | Fat digestion and absorption | -0.34483 | 0.746159 |
| hsa05166 | Human T-cell leukemia virus 1 infection | 0.362206 | 0.748103 |
| hsa04961 | Endocrine and other factor-regulated calcium reabsorption | -0.30672 | 0.750085 |
| hsa00330 | Arginine and proline metabolism | -0.29239 | 0.753087 |
| hsa05131 | Shigellosis | -0.2762 | 0.758513 |
| hsa04964 | Proximal tubule bicarbonate reclamation | -0.37373 | 0.761344 |
| hsa00565 | Ether lipid metabolism | -0.31677 | 0.761943 |
| hsa00591 | Linoleic acid metabolism | -0.34038 | 0.765212 |
| hsa04612 | Antigen processing and presentation | 0.390975 | 0.765729 |
| hsa00062 | Fatty acid elongation | -0.32668 | 0.771095 |
| hsa04933 | AGE-RAGE signaling pathway in diabetic complications | 0.372486 | 0.772669 |
| hsa04930 | Type II diabetes mellitus | -0.31099 | 0.773206 |
| hsa04130 | SNARE interactions in vesicular transport | -0.2989 | 0.773287 |
| hsa04922 | Glucagon signaling pathway | 0.391959 | 0.773813 |
| hsa00512 | Mucin type O-glycan biosynthesis | 0.450379 | 0.774485 |
| hsa04726 | Serotonergic synapse | 0.392421 | 0.776225 |
| hsa05014 | Amyotrophic lateral sclerosis (ALS) | -0.2958 | 0.780932 |
| hsa05134 | Legionellosis | 0.399129 | 0.783178 |
| hsa05223 | Non-small cell lung cancer | -0.26566 | 0.787593 |
| hsa05210 | Colorectal cancer | 0.383347 | 0.7881 |
| hsa04927 | Cortisol synthesis and secretion | 0.410666 | 0.789725 |
| hsa00260 | Glycine. serine and threonine metabolism | -0.28826 | 0.793553 |
| hsa04024 | cAMP signaling pathway | 0.363817 | 0.793734 |
| hsa05020 | Prion diseases | 0.437552 | 0.801005 |
| hsa04072 | Phospholipase D signaling pathway | -0.22529 | 0.817268 |
| hsa05016 | Huntington disease | 0.353922 | 0.834382 |
| hsa04014 | Ras signaling pathway | -0.22078 | 0.839485 |
| hsa04662 | B cell receptor signaling pathway | -0.24748 | 0.861469 |
| hsa00534 | Glycosaminoglycan biosynthesis | 0.464094 | 0.868407 |
| hsa05202 | Transcriptional misregulation in cancer | 0.353109 | 0.869555 |
| hsa00650 | Butanoate metabolism | 0.454009 | 0.871159 |
| hsa04970 | Salivary secretion | 0.381032 | 0.874355 |
| hsa05412 | Arrhythmogenic right ventricular cardiomyopathy (ARVC) | 0.387468 | 0.874524 |
| hsa04010 | MAPK signaling pathway | 0.334591 | 0.879304 |
| hsa04625 | C-type lectin receptor signaling pathway | 0.358793 | 0.881546 |
| hsa04012 | ErbB signaling pathway | 0.371266 | 0.883464 |
| hsa04742 | Taste transduction | 0.390851 | 0.892855 |
| hsa05146 | Amoebiasis | 0.361196 | 0.893064 |
| hsa05140 | Leishmaniasis | 0.379072 | 0.895061 |
| hsa03060 | Protein export | -0.28103 | 0.910718 |
| hsa04015 | Rap1 signaling pathway | -0.20267 | 0.911006 |
| hsa05219 | Bladder cancer | 0.400017 | 0.912963 |
| hsa05214 | Glioma | -0.22907 | 0.918144 |
| hsa00640 | Propanoate metabolism | -0.27244 | 0.921297 |
| hsa04979 | Cholesterol metabolism | -0.24726 | 0.921476 |
| hsa04724 | Glutamatergic synapse | -0.22222 | 0.922225 |
| hsa04152 | AMPK signaling pathway | -0.20242 | 0.922903 |
| hsa00511 | Other glycan degradation | -0.3178 | 0.926515 |
| hsa04614 | Renin-angiotensin system | -0.29006 | 0.930102 |
| hsa05220 | Chronic myeloid leukemia | -0.23752 | 0.932608 |
| hsa01200 | Carbon metabolism | -0.23131 | 0.933797 |
| hsa03410 | Base excision repair | -0.26702 | 0.93488 |
| hsa03430 | Mismatch repair | -0.30309 | 0.942216 |
| hsa04392 | Hippo signaling pathway | -0.29711 | 0.943634 |
| hsa00603 | Glycosphingolipid biosynthesis | -0.34054 | 0.944477 |
| hsa00270 | Cysteine and methionine metabolism | -0.2598 | 0.948764 |
| hsa05340 | Primary immunodeficiency | -0.27492 | 0.948887 |
| hsa04721 | Synaptic vesicle cycle | -0.25319 | 0.949743 |
| hsa04150 | mTOR signaling pathway | -0.20737 | 0.951047 |
| hsa05221 | Acute myeloid leukemia | -0.24077 | 0.957366 |
| hsa04114 | Oocyte meiosis | -0.21483 | 0.957922 |
| hsa04066 | HIF-1 signaling pathway | -0.21967 | 0.958378 |
| hsa05222 | Small cell lung cancer | -0.21764 | 0.961414 |
| hsa04925 | Aldosterone synthesis and secretion | 0.353592 | 0.970059 |
| hsa00010 | Glycolysis / Gluconeogenesis | 0.374964 | 0.980552 |
| hsa00020 | Citrate cycle (TCA cycle) | 0.420183 | 0.989464 |
| hsa04550 | Signaling pathways regulating pluripotency of stem cells | 0.342819 | 0.990646 |
| hsa03022 | Basal transcription factors | -0.116 | 0.999863 |
| hsa03040 | Spliceosome | 0.106225 | 0.999954 |

***COPD, moderate cores vs CT***

| geneSet | description | ES | FDR |
| --- | --- | --- | --- |
| hsa00982 | Drug metabolism | 0.758153 | **0** |
| hsa05204 | Chemical carcinogenesis | 0.737791 | **0** |
| hsa00980 | Metabolism of xenobiotics by cytochrome P450 | 0.73791 | **0** |
| hsa00040 | Pentose and glucuronate interconversions | 0.799666 | **0** |
| hsa00860 | Porphyrin and chlorophyll metabolism | 0.778444 | **0** |
| hsa00053 | Ascorbate and aldarate metabolism | 0.845577 | **0** |
| hsa00830 | Retinol metabolism | 0.692917 | **0.00072** |
| hsa00983 | Drug metabolism | 0.674905 | **0.00084** |
| hsa04672 | Intestinal immune network for IgA production | 0.698509 | **0.001493** |
| hsa05144 | Malaria | 0.693454 | **0.001679** |
| hsa00140 | Steroid hormone biosynthesis | 0.672358 | **0.003511** |
| hsa05143 | African trypanosomiasis | 0.693066 | **0.007486** |
| hsa05323 | Rheumatoid arthritis | 0.602005 | **0.020601** |
| hsa04740 | Olfactory transduction | 0.554945 | **0.021528** |
| hsa03008 | Ribosome biogenesis in eukaryotes | -0.50622 | **0.023049** |
| hsa04657 | IL-17 signaling pathway | 0.59534 | **0.027033** |
| hsa04940 | Type I diabetes mellitus | 0.640542 | **0.030538** |
| hsa04611 | Platelet activation | -0.44993 | **0.04264** |
| hsa05310 | Asthma | 0.662619 | **0.06662** |
| hsa00650 | Butanoate metabolism | 0.667529 | **0.090903** |
| hsa04060 | Cytokine-cytokine receptor interaction | 0.52102 | **0.094205** |
| hsa04915 | Estrogen signaling pathway | 0.548097 | **0.095875** |
| hsa05330 | Allograft rejection | 0.590044 | 0.178262 |
| hsa00601 | Glycosphingolipid biosynthesis | 0.623793 | 0.240642 |
| hsa05332 | Graft-versus-host disease | 0.566176 | 0.272558 |
| hsa05321 | Inflammatory bowel disease (IBD) | 0.528539 | 0.29933 |
| hsa04350 | TGF-beta signaling pathway | 0.507464 | 0.314298 |
| hsa05016 | Huntington disease | 0.487268 | 0.318688 |
| hsa05322 | Systemic lupus erythematosus | 0.530472 | 0.321446 |
| hsa00591 | Linoleic acid metabolism | 0.595913 | 0.324841 |
| hsa00670 | One carbon pool by folate | -0.54234 | 0.379922 |
| hsa04650 | Natural killer cell mediated cytotoxicity | -0.36031 | 0.384916 |
| hsa04512 | ECM-receptor interaction | -0.37143 | 0.391172 |
| hsa00604 | Glycosphingolipid biosynthesis | -0.53306 | 0.393298 |
| hsa00410 | beta-Alanine metabolism | 0.568544 | 0.395046 |
| hsa04370 | VEGF signaling pathway | -0.36736 | 0.397859 |
| hsa00532 | Glycosaminoglycan biosynthesis | -0.53667 | 0.401511 |
| hsa04966 | Collecting duct acid secretion | 0.586314 | 0.403174 |
| hsa05150 | Staphylococcus aureus infection | 0.522796 | 0.409744 |
| hsa00515 | Mannose type O-glycan biosynthesis | -0.50821 | 0.412114 |
| hsa00220 | Arginine biosynthesis | -0.54205 | 0.416608 |
| hsa05142 | Chagas disease (American trypanosomiasis) | 0.479304 | 0.421677 |
| hsa05320 | Autoimmune thyroid disease | 0.516646 | 0.424832 |
| hsa03440 | Homologous recombination | -0.47379 | 0.426404 |
| hsa00310 | Lysine degradation | -0.36858 | 0.42698 |
| hsa00480 | Glutathione metabolism | 0.517697 | 0.436803 |
| hsa04730 | Long-term depression | -0.40567 | 0.439465 |
| hsa00350 | Tyrosine metabolism | 0.553751 | 0.44053 |
| hsa04640 | Hematopoietic cell lineage | 0.473694 | 0.444791 |
| hsa00360 | Phenylalanine metabolism | 0.640782 | 0.45654 |
| hsa05100 | Bacterial invasion of epithelial cells | -0.35016 | 0.457026 |
| hsa03450 | Non-homologous end-joining | -0.51026 | 0.474192 |
| hsa04062 | Chemokine signaling pathway | 0.452006 | 0.476556 |
| hsa01040 | Biosynthesis of unsaturated fatty acids | -0.41786 | 0.480281 |
| hsa04610 | Complement and coagulation cascades | 0.49356 | 0.485915 |
| hsa00010 | Glycolysis / Gluconeogenesis | 0.495758 | 0.490668 |
| hsa00071 | Fatty acid degradation | 0.515348 | 0.497026 |
| hsa00072 | Synthesis and degradation of ketone bodies | 0.655293 | 0.511798 |
| hsa03010 | Ribosome | -0.2946 | 0.522185 |
| hsa00970 | Aminoacyl-tRNA biosynthesis | -0.41117 | 0.534734 |
| hsa04666 | Fc gamma R-mediated phagocytosis | -0.28553 | 0.648503 |
| hsa00730 | Thiamine metabolism | 0.608061 | 0.653999 |
| hsa04622 | RIG-I-like receptor signaling pathway | -0.31065 | 0.65478 |
| hsa04015 | Rap1 signaling pathway | -0.27172 | 0.659198 |
| hsa01523 | Antifolate resistance | -0.38052 | 0.66126 |
| hsa01524 | Platinum drug resistance | 0.457619 | 0.666917 |
| hsa04514 | Cell adhesion molecules (CAMs) | 0.440915 | 0.669424 |
| hsa05340 | Primary immunodeficiency | 0.507743 | 0.68199 |
| hsa03460 | Fanconi anemia pathway | -0.32419 | 0.682247 |
| hsa05131 | Shigellosis | -0.3053 | 0.687317 |
| hsa00450 | Selenocompound metabolism | -0.43069 | 0.687515 |
| hsa05014 | Amyotrophic lateral sclerosis (ALS) | -0.33073 | 0.688363 |
| hsa04659 | Th17 cell differentiation | 0.441368 | 0.696542 |
| hsa00052 | Galactose metabolism | -0.35495 | 0.731206 |
| hsa00340 | Histidine metabolism | 0.540225 | 0.735732 |
| hsa04072 | Phospholipase D signaling pathway | -0.26812 | 0.741491 |
| hsa00630 | Glyoxylate and dicarboxylate metabolism | -0.36033 | 0.743655 |
| hsa00561 | Glycerolipid metabolism | -0.29911 | 0.752315 |
| hsa04392 | Hippo signaling pathway | -0.33875 | 0.757193 |
| hsa04744 | Phototransduction | 0.604299 | 0.762214 |
| hsa00590 | Arachidonic acid metabolism | 0.459866 | 0.770103 |
| hsa05031 | Amphetamine addiction | 0.463922 | 0.771841 |
| hsa00600 | Sphingolipid metabolism | 0.478682 | 0.783893 |
| hsa04270 | Vascular smooth muscle contraction | -0.25372 | 0.817642 |
| hsa04130 | SNARE interactions in vesicular transport | -0.29926 | 0.822663 |
| hsa00770 | Pantothenate and CoA biosynthesis | -0.36179 | 0.828341 |
| hsa05414 | Dilated cardiomyopathy (DCM) | -0.2604 | 0.830667 |
| hsa00640 | Propanoate metabolism | -0.33393 | 0.83803 |
| hsa04961 | Endocrine and other factor-regulated calcium reabsorption | -0.2916 | 0.840845 |
| hsa00380 | Tryptophan metabolism | -0.31737 | 0.843526 |
| hsa04510 | Focal adhesion | -0.25036 | 0.843589 |
| hsa04742 | Taste transduction | -0.27627 | 0.846643 |
| hsa04914 | Progesterone-mediated oocyte maturation | -0.26078 | 0.846982 |
| hsa00510 | N-Glycan biosynthesis | -0.28049 | 0.85382 |
| hsa04621 | NOD-like receptor signaling pathway | -0.2192 | 0.864976 |
| hsa04668 | TNF signaling pathway | 0.423199 | 0.865642 |
| hsa04630 | JAK-STAT signaling pathway | 0.407333 | 0.866719 |
| hsa04710 | Circadian rhythm | 0.477807 | 0.868445 |
| hsa00100 | Steroid biosynthesis | 0.522321 | 0.871084 |
| hsa00534 | Glycosaminoglycan biosynthesis | 0.523563 | 0.877518 |
| hsa05030 | Cocaine addiction | 0.473606 | 0.882426 |
| hsa05033 | Nicotine addiction | 0.584342 | 0.893454 |
| hsa05416 | Viral myocarditis | 0.439756 | 0.896081 |
| hsa05223 | Non-small cell lung cancer | -0.24945 | 0.902835 |
| hsa04972 | Pancreatic secretion | 0.424838 | 0.905089 |
| hsa00330 | Arginine and proline metabolism | -0.24822 | 0.907396 |
| hsa04726 | Serotonergic synapse | 0.421222 | 0.91102 |
| hsa05418 | Fluid shear stress and atherosclerosis | 0.410926 | 0.911138 |
| hsa03015 | mRNA surveillance pathway | -0.2246 | 0.918048 |
| hsa04924 | Renin secretion | 0.440534 | 0.924944 |
| hsa05224 | Breast cancer | -0.20641 | 0.925452 |
| hsa00603 | Glycosphingolipid biosynthesis | -0.35506 | 0.925958 |
| hsa05132 | Salmonella infection | 0.409395 | 0.93022 |
| hsa00520 | Amino sugar and nucleotide sugar metabolism | -0.24766 | 0.934138 |
| hsa04964 | Proximal tubule bicarbonate reclamation | -0.3528 | 0.935118 |
| hsa04658 | Th1 and Th2 cell differentiation | 0.409996 | 0.935774 |
| hsa04810 | Regulation of actin cytoskeleton | -0.20034 | 0.942699 |
| hsa05133 | Pertussis | 0.420178 | 0.944388 |
| hsa01212 | Fatty acid metabolism | -0.25313 | 0.945423 |
| hsa03030 | DNA replication | -0.28127 | 0.946573 |
| hsa04960 | Aldosterone-regulated sodium reabsorption | -0.28551 | 0.947778 |
| hsa04664 | Fc epsilon RI signaling pathway | -0.24271 | 0.948904 |
| hsa05214 | Glioma | -0.20566 | 0.950898 |
| hsa00620 | Pyruvate metabolism | -0.2292 | 0.952171 |
| hsa00240 | Pyrimidine metabolism | -0.18646 | 0.953217 |
| hsa05212 | Pancreatic cancer | -0.20711 | 0.953555 |
| hsa04725 | Cholinergic synapse | -0.1913 | 0.955664 |
| hsa05222 | Small cell lung cancer | -0.22443 | 0.958969 |
| hsa00512 | Mucin type O-glycan biosynthesis | 0.469465 | 0.963387 |
| hsa05220 | Chronic myeloid leukemia | -0.21243 | 0.963852 |
| hsa04978 | Mineral absorption | -0.23697 | 0.965181 |
| hsa00790 | Folate biosynthesis | -0.31425 | 0.966048 |
| hsa01210 | 2-Oxocarboxylic acid metabolism | -0.27419 | 0.967375 |
| hsa03050 | Proteasome | -0.23561 | 0.968089 |
| hsa05146 | Amoebiasis | 0.396214 | 0.969042 |
| hsa04979 | Cholesterol metabolism | -0.26327 | 0.970451 |
| hsa04066 | HIF-1 signaling pathway | -0.2302 | 0.97079 |
| hsa05219 | Bladder cancer | 0.447522 | 0.972233 |
| hsa04152 | AMPK signaling pathway | -0.16751 | 0.972478 |
| hsa01230 | Biosynthesis of amino acids | -0.23873 | 0.976765 |
| hsa04150 | mTOR signaling pathway | -0.18007 | 0.978304 |
| hsa00062 | Fatty acid elongation | -0.22626 | 0.980761 |
| hsa04114 | Oocyte meiosis | -0.18933 | 0.98712 |
| hsa05020 | Prion diseases | 0.447674 | 0.988502 |
| hsa04330 | Notch signaling pathway | -0.18323 | 0.993194 |
| hsa03430 | Mismatch repair | -0.15502 | 0.99717 |
| hsa03410 | Base excision repair | -0.18502 | 0.998525 |
| hsa04110 | Cell cycle | -0.19053 | 0.99869 |
| hsa03040 | Spliceosome | 0.09959 | 0.999996 |

***COPD, severe cores vs CT***

| geneSet | description | ES | FDR |
| --- | --- | --- | --- |
| hsa04740 | Olfactory transduction | 0.809581 | **0** |
| hsa05144 | Malaria | 0.734745 | **0** |
| hsa05320 | Autoimmune thyroid disease | 0.750552 | **0** |
| hsa05332 | Graft-versus-host disease | 0.742318 | **0** |
| hsa04672 | Intestinal immune network for IgA production | 0.727751 | **0** |
| hsa05323 | Rheumatoid arthritis | 0.643199 | **0** |
| hsa04060 | Cytokine-cytokine receptor interaction | 0.553773 | **0.00031** |
| hsa05143 | African trypanosomiasis | 0.731447 | **0.000354** |
| hsa05330 | Allograft rejection | 0.710236 | **0.00055** |
| hsa05310 | Asthma | 0.737526 | **0.001276** |
| hsa04940 | Type I diabetes mellitus | 0.687438 | **0.001403** |
| hsa05321 | Inflammatory bowel disease (IBD) | 0.623439 | **0.001444** |
| hsa04630 | JAK-STAT signaling pathway | 0.548703 | **0.00254** |
| hsa05132 | Salmonella infection | 0.565876 | **0.003773** |
| hsa04668 | TNF signaling pathway | 0.54702 | **0.004622** |
| hsa04623 | Cytosolic DNA-sensing pathway | 0.590555 | **0.005468** |
| hsa04512 | ECM-receptor interaction | -0.55209 | **0.005728** |
| hsa05150 | Staphylococcus aureus infection | 0.596211 | **0.012672** |
| hsa05416 | Viral myocarditis | 0.581681 | **0.015086** |
| hsa04611 | Platelet activation | -0.48629 | **0.017901** |
| hsa04657 | IL-17 signaling pathway | 0.524581 | **0.025631** |
| hsa05168 | Herpes simplex infection | 0.46836 | **0.042449** |
| hsa04620 | Toll-like receptor signaling pathway | 0.499053 | **0.043371** |
| hsa05164 | Influenza A | 0.472531 | **0.043457** |
| hsa04659 | Th17 cell differentiation | 0.4936 | **0.045982** |
| hsa05142 | Chagas disease (American trypanosomiasis) | 0.496892 | **0.046007** |
| hsa04350 | TGF-beta signaling pathway | 0.50786 | **0.047774** |
| hsa04514 | Cell adhesion molecules (CAMs) | 0.471309 | **0.054699** |
| hsa04966 | Collecting duct acid secretion | 0.621892 | **0.058237** |
| hsa05167 | Kaposi sarcoma-associated herpesvirus infection | 0.450063 | **0.063851** |
| hsa05322 | Systemic lupus erythematosus | 0.535378 | **0.065317** |
| hsa04640 | Hematopoietic cell lineage | 0.480236 | **0.066374** |
| hsa00052 | Galactose metabolism | -0.60361 | **0.066592** |
| hsa00220 | Arginine biosynthesis | -0.64304 | **0.074946** |
| hsa04110 | Cell cycle | -0.43203 | **0.082107** |
| hsa00532 | Glycosaminoglycan biosynthesis | -0.62102 | **0.088994** |
| hsa00790 | Folate biosynthesis | -0.62912 | **0.094232** |
| hsa05163 | Human cytomegalovirus infection | 0.424289 | 0.103202 |
| hsa05140 | Leishmaniasis | 0.478845 | 0.112383 |
| hsa04062 | Chemokine signaling pathway | 0.420597 | 0.137667 |
| hsa00350 | Tyrosine metabolism | -0.53269 | 0.149097 |
| hsa05162 | Measles | 0.436117 | 0.152675 |
| hsa04612 | Antigen processing and presentation | 0.469602 | 0.156331 |
| hsa01210 | 2-Oxocarboxylic acid metabolism | -0.59987 | 0.156635 |
| hsa05031 | Amphetamine addiction | 0.483053 | 0.168025 |
| hsa04658 | Th1 and Th2 cell differentiation | 0.451844 | 0.171317 |
| hsa00730 | Thiamine metabolism | -0.62991 | 0.174572 |
| hsa01040 | Biosynthesis of unsaturated fatty acids | -0.52277 | 0.176698 |
| hsa03008 | Ribosome biogenesis in eukaryotes | -0.41289 | 0.182711 |
| hsa04914 | Progesterone-mediated oocyte maturation | -0.41309 | 0.194244 |
| hsa05152 | Tuberculosis | 0.401017 | 0.194753 |
| hsa05020 | Prion diseases | 0.514789 | 0.196576 |
| hsa05133 | Pertussis | 0.441534 | 0.199684 |
| hsa04650 | Natural killer cell mediated cytotoxicity | 0.417261 | 0.204639 |
| hsa04970 | Salivary secretion | -0.39027 | 0.243284 |
| hsa03440 | Homologous recombination | -0.4795 | 0.244601 |
| hsa03460 | Fanconi anemia pathway | -0.42266 | 0.250974 |
| hsa00670 | One carbon pool by folate | -0.51967 | 0.252168 |
| hsa00515 | Mannose type O-glycan biosynthesis | -0.56174 | 0.254107 |
| hsa00051 | Fructose and mannose metabolism | -0.50717 | 0.254503 |
| hsa04270 | Vascular smooth muscle contraction | -0.369 | 0.254997 |
| hsa04923 | Regulation of lipolysis in adipocytes | 0.477872 | 0.25725 |
| hsa05160 | Hepatitis C | 0.405495 | 0.258519 |
| hsa00010 | Glycolysis / Gluconeogenesis | -0.40142 | 0.259894 |
| hsa04064 | NF-kappa B signaling pathway | 0.417275 | 0.260788 |
| hsa04510 | Focal adhesion | -0.34099 | 0.262299 |
| hsa04330 | Notch signaling pathway | -0.42352 | 0.268322 |
| hsa00603 | Glycosphingolipid biosynthesis | -0.58133 | 0.274416 |
| hsa00480 | Glutathione metabolism | -0.42031 | 0.274564 |
| hsa00310 | Lysine degradation | -0.39572 | 0.279093 |
| hsa05169 | Epstein-Barr virus infection | 0.378615 | 0.285167 |
| hsa05161 | Hepatitis B | 0.390618 | 0.289949 |
| hsa00533 | Glycosaminoglycan biosynthesis | -0.56726 | 0.296708 |
| hsa04666 | Fc gamma R-mediated phagocytosis | -0.36106 | 0.299972 |
| hsa04742 | Taste transduction | 0.444979 | 0.316853 |
| hsa05340 | Primary immunodeficiency | 0.488883 | 0.318376 |
| hsa04726 | Serotonergic synapse | 0.409326 | 0.321047 |
| hsa04610 | Complement and coagulation cascades | 0.420789 | 0.321421 |
| hsa05166 | Human T-cell leukemia virus 1 infection | 0.362581 | 0.323177 |
| hsa04928 | Parathyroid hormone synthesis. secretion and action | 0.400839 | 0.332795 |
| hsa05030 | Cocaine addiction | 0.465061 | 0.335953 |
| hsa04660 | T cell receptor signaling pathway | 0.395943 | 0.336748 |
| hsa04918 | Thyroid hormone synthesis | -0.37288 | 0.336838 |
| hsa00980 | Metabolism of xenobiotics by cytochrome P450 | -0.36084 | 0.337481 |
| hsa01230 | Biosynthesis of amino acids | -0.36847 | 0.33899 |
| hsa05134 | Legionellosis | 0.431082 | 0.344762 |
| hsa00040 | Pentose and glucuronate interconversions | -0.43852 | 0.346041 |
| hsa04625 | C-type lectin receptor signaling pathway | 0.385189 | 0.354285 |
| hsa00561 | Glycerolipid metabolism | -0.38366 | 0.357502 |
| hsa04380 | Osteoclast differentiation | 0.375886 | 0.359211 |
| hsa00630 | Glyoxylate and dicarboxylate metabolism | -0.42632 | 0.375822 |
| hsa04530 | Tight junction | 0.364081 | 0.376329 |
| hsa00072 | Synthesis and degradation of ketone bodies | 0.587897 | 0.377678 |
| hsa04621 | NOD-like receptor signaling pathway | 0.362998 | 0.378677 |
| hsa04622 | RIG-I-like receptor signaling pathway | 0.408014 | 0.381349 |
| hsa03050 | Proteasome | -0.38951 | 0.384053 |
| hsa05033 | Nicotine addiction | -0.54588 | 0.384238 |
| hsa04710 | Circadian rhythm | 0.470817 | 0.387299 |
| hsa04932 | Non-alcoholic fatty liver disease (NAFLD) | 0.36239 | 0.395105 |
| hsa04964 | Proximal tubule bicarbonate reclamation | -0.47931 | 0.402493 |
| hsa00983 | Drug metabolism | -0.34893 | 0.407063 |
| hsa03450 | Non-homologous end-joining | -0.53393 | 0.408954 |
| hsa00140 | Steroid hormone biosynthesis | -0.36098 | 0.410347 |
| hsa04015 | Rap1 signaling pathway | -0.29415 | 0.419496 |
| hsa04080 | Neuroactive ligand-receptor interaction | 0.355468 | 0.422542 |
| hsa04010 | MAPK signaling pathway | 0.337281 | 0.424927 |
| hsa04974 | Protein digestion and absorption | -0.34071 | 0.427063 |
| hsa05212 | Pancreatic cancer | -0.33558 | 0.430018 |
| hsa04114 | Oocyte meiosis | -0.31531 | 0.435731 |
| hsa05223 | Non-small cell lung cancer | -0.33827 | 0.438282 |
| hsa00830 | Retinol metabolism | -0.35077 | 0.443439 |
| hsa00512 | Mucin type O-glycan biosynthesis | -0.39515 | 0.4512 |
| hsa04924 | Renin secretion | 0.399842 | 0.471667 |
| hsa05206 | MicroRNAs in cancer | -0.28045 | 0.474904 |
| hsa00270 | Cysteine and methionine metabolism | -0.36704 | 0.476141 |
| hsa05100 | Bacterial invasion of epithelial cells | -0.33145 | 0.477237 |
| hsa00120 | Primary bile acid biosynthesis | 0.544662 | 0.480125 |
| hsa04724 | Glutamatergic synapse | -0.31382 | 0.482559 |
| hsa04933 | AGE-RAGE signaling pathway in diabetic complications | 0.366623 | 0.483406 |
| hsa00360 | Phenylalanine metabolism | -0.48507 | 0.485508 |
| hsa04917 | Prolactin signaling pathway | 0.394619 | 0.51578 |
| hsa04730 | Long-term depression | -0.3375 | 0.523876 |
| hsa05222 | Small cell lung cancer | -0.29595 | 0.535296 |
| hsa04927 | Cortisol synthesis and secretion | 0.392872 | 0.535721 |
| hsa00510 | N-Glycan biosynthesis | -0.34065 | 0.537087 |
| hsa04072 | Phospholipase D signaling pathway | -0.28569 | 0.539925 |
| hsa03410 | Base excision repair | -0.3597 | 0.562209 |
| hsa04217 | Necroptosis | 0.342169 | 0.565632 |
| hsa01200 | Carbon metabolism | -0.28868 | 0.568664 |
| hsa00500 | Starch and sucrose metabolism | -0.3661 | 0.571138 |
| hsa00982 | Drug metabolism | -0.30794 | 0.571616 |
| hsa04370 | VEGF signaling pathway | -0.3201 | 0.57329 |
| hsa04022 | cGMP-PKG signaling pathway | -0.26899 | 0.590253 |
| hsa05130 | Pathogenic Escherichia coli infection | -0.30846 | 0.607506 |
| hsa00410 | beta-Alanine metabolism | -0.35498 | 0.610598 |
| hsa05214 | Glioma | -0.30397 | 0.611371 |
| hsa00330 | Arginine and proline metabolism | -0.32492 | 0.611727 |
| hsa03030 | DNA replication | -0.3421 | 0.612163 |
| hsa00531 | Glycosaminoglycan degradation | -0.40376 | 0.612371 |
| hsa05219 | Bladder cancer | 0.391986 | 0.613093 |
| hsa05120 | Epithelial cell signaling in Helicobacter pylori infection | 0.368538 | 0.614259 |
| hsa00650 | Butanoate metabolism | 0.445351 | 0.619008 |
| hsa05170 | Human immunodeficiency virus 1 infection | 0.312379 | 0.621067 |
| hsa04213 | Longevity regulating pathway | 0.372382 | 0.625404 |
| hsa04978 | Mineral absorption | 0.388477 | 0.627888 |
| hsa05110 | Vibrio cholerae infection | 0.382352 | 0.628243 |
| hsa04922 | Glucagon signaling pathway | 0.351656 | 0.633306 |
| hsa04392 | Hippo signaling pathway | -0.35569 | 0.642926 |
| hsa04973 | Carbohydrate digestion and absorption | -0.3388 | 0.644542 |
| hsa00053 | Ascorbate and aldarate metabolism | -0.37548 | 0.648349 |
| hsa02010 | ABC transporters | -0.3399 | 0.659996 |
| hsa05414 | Dilated cardiomyopathy (DCM) | -0.28565 | 0.66124 |
| hsa00590 | Arachidonic acid metabolism | -0.29677 | 0.662371 |
| hsa00514 | Other types of O-glycan biosynthesis | -0.36966 | 0.663746 |
| hsa04115 | p53 signaling pathway | -0.28507 | 0.666827 |
| hsa00604 | Glycosphingolipid biosynthesis | -0.41838 | 0.667688 |
| hsa04670 | Leukocyte transendothelial migration | 0.332926 | 0.684726 |
| hsa00534 | Glycosaminoglycan biosynthesis | 0.443405 | 0.692006 |
| hsa05224 | Breast cancer | -0.25454 | 0.693298 |
| hsa05418 | Fluid shear stress and atherosclerosis | 0.314414 | 0.697866 |
| hsa00592 | alpha-Linolenic acid metabolism | -0.35871 | 0.706728 |
| hsa05131 | Shigellosis | -0.2818 | 0.709781 |
| hsa00860 | Porphyrin and chlorophyll metabolism | -0.3118 | 0.71373 |
| hsa04218 | Cellular senescence | -0.24422 | 0.718427 |
| hsa04261 | Adrenergic signaling in cardiomyocytes | -0.25292 | 0.720465 |
| hsa01522 | Endocrine resistance | -0.26036 | 0.740272 |
| hsa04723 | Retrograde endocannabinoid signaling | -0.24488 | 0.780442 |
| hsa00260 | Glycine. serine and threonine metabolism | -0.29913 | 0.784884 |
| hsa00564 | Glycerophospholipid metabolism | -0.25959 | 0.787766 |
| hsa00900 | Terpenoid backbone biosynthesis | 0.41204 | 0.788405 |
| hsa00380 | Tryptophan metabolism | -0.30675 | 0.794561 |
| hsa05202 | Transcriptional misregulation in cancer | 0.303532 | 0.796432 |
| hsa05210 | Colorectal cancer | 0.327611 | 0.797196 |
| hsa04961 | Endocrine and other factor-regulated calcium reabsorption | -0.29728 | 0.79813 |
| hsa04020 | Calcium signaling pathway | 0.300857 | 0.806331 |
| hsa05032 | Morphine addiction | 0.340846 | 0.807242 |
| hsa05145 | Toxoplasmosis | 0.312645 | 0.808522 |
| hsa04915 | Estrogen signaling pathway | 0.31039 | 0.813139 |
| hsa05146 | Amoebiasis | 0.31982 | 0.814137 |
| hsa00450 | Selenocompound metabolism | -0.36911 | 0.819952 |
| hsa04024 | cAMP signaling pathway | 0.292533 | 0.831922 |
| hsa04921 | Oxytocin signaling pathway | 0.301769 | 0.838239 |
| hsa04913 | Ovarian steroidogenesis | 0.357108 | 0.841634 |
| hsa05216 | Thyroid cancer | 0.361137 | 0.858505 |
| hsa04960 | Aldosterone-regulated sodium reabsorption | -0.30191 | 0.859523 |
| hsa05225 | Hepatocellular carcinoma | -0.22362 | 0.863598 |
| hsa05215 | Prostate cancer | -0.23823 | 0.863673 |
| hsa05220 | Chronic myeloid leukemia | -0.24173 | 0.864195 |
| hsa04975 | Fat digestion and absorption | -0.32874 | 0.864441 |
| hsa00240 | Pyrimidine metabolism | -0.23727 | 0.864526 |
| hsa03430 | Mismatch repair | -0.31905 | 0.868713 |
| hsa05165 | Human papillomavirus infection | -0.20509 | 0.870697 |
| hsa04925 | Aldosterone synthesis and secretion | 0.31457 | 0.873369 |
| hsa01212 | Fatty acid metabolism | -0.26292 | 0.873993 |
| hsa04727 | GABAergic synapse | -0.25207 | 0.874725 |
| hsa00062 | Fatty acid elongation | -0.28883 | 0.878784 |
| hsa00250 | Alanine. aspartate and glutamate metabolism | -0.2917 | 0.879656 |
| hsa05221 | Acute myeloid leukemia | -0.24879 | 0.88141 |
| hsa05410 | Hypertrophic cardiomyopathy (HCM) | -0.24305 | 0.88158 |
| hsa04919 | Thyroid hormone signaling pathway | -0.2251 | 0.882197 |
| hsa05200 | Pathways in cancer | 0.262943 | 0.882284 |
| hsa04725 | Cholinergic synapse | -0.23896 | 0.883326 |
| hsa05231 | Choline metabolism in cancer | -0.22634 | 0.885166 |
| hsa05014 | Amyotrophic lateral sclerosis (ALS) | -0.25724 | 0.885361 |
| hsa04550 | Signaling pathways regulating pluripotency of stem cells | 0.294708 | 0.886514 |
| hsa04810 | Regulation of actin cytoskeleton | -0.20588 | 0.886738 |
| hsa04145 | Phagosome | 0.287006 | 0.887423 |
| hsa04664 | Fc epsilon RI signaling pathway | -0.24688 | 0.891285 |
| hsa04721 | Synaptic vesicle cycle | -0.25012 | 0.893161 |
| hsa04911 | Insulin secretion | 0.323088 | 0.893406 |
| hsa04662 | B cell receptor signaling pathway | 0.311658 | 0.89516 |
| hsa00030 | Pentose phosphate pathway | 0.362328 | 0.900136 |
| hsa04972 | Pancreatic secretion | 0.308439 | 0.900838 |
| hsa05230 | Central carbon metabolism in cancer | -0.23369 | 0.905862 |
| hsa05204 | Chemical carcinogenesis | 0.308318 | 0.906136 |
| hsa04728 | Dopaminergic synapse | 0.288033 | 0.91437 |
| hsa03010 | Ribosome | 0.279814 | 0.91483 |
| hsa04977 | Vitamin digestion and absorption | 0.371723 | 0.91642 |
| hsa04930 | Type II diabetes mellitus | 0.34264 | 0.919382 |
| hsa04211 | Longevity regulating pathway | 0.297348 | 0.924781 |
| hsa04151 | PI3K-Akt signaling pathway | 0.260257 | 0.925109 |
| hsa00565 | Ether lipid metabolism | 0.346674 | 0.93039 |
| hsa04979 | Cholesterol metabolism | -0.2557 | 0.932761 |
| hsa04071 | Sphingolipid signaling pathway | -0.20835 | 0.935307 |
| hsa04150 | mTOR signaling pathway | -0.20175 | 0.936576 |
| hsa04614 | Renin-angiotensin system | 0.371898 | 0.938046 |
| hsa03320 | PPAR signaling pathway | 0.30266 | 0.938611 |
| hsa04216 | Ferroptosis | 0.328763 | 0.940504 |
| hsa04310 | Wnt signaling pathway | 0.270516 | 0.941667 |
| hsa00100 | Steroid biosynthesis | 0.378717 | 0.945515 |
| hsa03015 | mRNA surveillance pathway | -0.21245 | 0.95086 |
| hsa04744 | Phototransduction | 0.404344 | 0.980181 |
| hsa04210 | Apoptosis | 0.262466 | 0.982729 |
| hsa04260 | Cardiac muscle contraction | -0.22132 | 0.983945 |
| hsa00591 | Linoleic acid metabolism | 0.356673 | 0.984242 |
| hsa04976 | Bile secretion | 0.29969 | 0.986784 |
| hsa05203 | Viral carcinogenesis | 0.25419 | 0.987539 |
| hsa00970 | Aminoacyl-tRNA biosynthesis | -0.25292 | 0.987852 |
| hsa04926 | Relaxin signaling pathway | 0.258419 | 0.990143 |
| hsa04920 | Adipocytokine signaling pathway | 0.296367 | 0.990164 |
| hsa04068 | FoxO signaling pathway | 0.257963 | 0.990284 |
| hsa00760 | Nicotinate and nicotinamide metabolism | 0.321333 | 0.991048 |
| hsa04520 | Adherens junction | 0.282954 | 0.995158 |
| hsa03060 | Protein export | -0.17596 | 0.996847 |
| hsa00190 | Oxidative phosphorylation | 0.274087 | 0.997269 |
| hsa04012 | ErbB signaling pathway | 0.275492 | 0.997461 |
| hsa04130 | SNARE interactions in vesicular transport | 0.150735 | 0.999341 |

# Supplementary Figures

**Figure E1** : Innate immune cell type enrichment scores comparison

Figure legend: LogFC of xCell enrichment scores of neutrophils, eosinophils and basophils in mild COPD cores, moderate COPD cores and severe COPD cores as compared with control cores (ns= non significant).
